# Chiral‐Encoded Pt‐Ir Surfaces as Apparent Spin Filter for Enhanced Oxygen Reduction

**DOI:** 10.1002/advs.75175

**Published:** 2026-04-10

**Authors:** Zikkawas Pasom, Krissanapat Yomthong, Sopon Butcha, Jonas Fransson, Chularat Wattanakit, Alexander Kuhn

**Affiliations:** ^1^ School of Energy Science and Engineering Vidyasirimedhi Institute of Science and Technology Rayong Thailand; ^2^ University of Bordeaux CNRS Bordeaux INP ISM UMR 5255 Pessac France; ^3^ Department of Physics and Astronomy Uppsala University Uppsala Sweden

**Keywords:** chiral‐encoded mesoporous metal, chiral‐induced spin selectivity, electrocatalysis, electrodeposition, oxygen reduction reaction

## Abstract

Chiral Induced Spin Selectivity (CISS) is a promising effect for enhancing the electrochemical oxygen reduction reaction (ORR), an essential process in clean energy technologies. So far, the concept has been validated mostly for surfaces modified with chiral organic layers; however, it remains unexplored for chiral metal matrices. Here, we study chiral‐encoded mesoporous Pt‐Ir alloys, bearing high selectivity when used for the analysis, separation, and synthesis of enantiomers. We demonstrate a very substantial difference in ORR activity for Pt‐Ir electrodes imprinted with molecules of opposite handedness. One enantiomorph catalyzes oxygen reduction, whereas its mirror image surprisingly acts as an inhibitor. This very unusual asymmetric reactivity is due to an interplay between the CISS effect and the adsorption of oxygen on the metal surface, leading to a break of symmetry in terms of spin interactions. Our findings open up promising perspectives for designing CISS‐based ORR catalysts, advancing the development of clean energy technologies.

## Graphical abstract

The Chiral‐Induced Spin Selectivity (CISS) effect is employed in chiral‐encoded mesoporous Platinum‐Iridium (Pt‐Ir) electrocatalysts to enhance the oxygen reduction reaction (ORR). A break in spin symmetry leads to a substantial antagonistic difference in activity (>200%) when comparing two enantiomorphs of the same electrode.

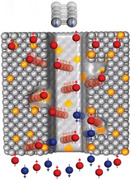

## 1 Introduction

Currently, clean energy sources, including solar [1, 2], wind [3, 4], tidal [5], and water [6], have attracted great interest as next‐generation renewable energy technologies with high efficiency [1, 7]. Therefore, developing and optimizing high‐performance energy conversion systems is crucial [8]. Among all the possible devices, fuel cells have drawn much attention as a promising alternative to traditional combustion‐based technologies, with the potential to provide clean and reliable power [9, 10, 11]. Fuel cells offer several advantages over conventional energy conversion systems, such as higher efficiency, lower emissions, and quiet operation [12, 13]. However, the involved oxygen reduction reaction (ORR) to water is often the rate‐limiting process [14, 15, 16] to produce electricity with an optimized power output [17, 18]. Thus, increasing the efficiency of ORR is critical for developing high‐performance low‐cost fuel cells [18, 19]. As ORR is a kinetically sluggish process, it requires specific catalysts to accelerate the reaction rate [20], but their high cost and scarcity often hinder the widespread use of fuel cell technologies [21]. Therefore, extensive research efforts currently focus on developing alternative catalytic systems that are more affordable and abundant [22].

In general, the electronic state of diatomic oxygen is a triplet state [23, 24], which converts into a singlet during ORR [25]. This spin‐forbidden transition is a significant challenge for achieving efficient four‐electron ORR. To overcome this obstacle, spin‐controlled catalysis has been proposed as a concept for improving ORR. One promising strategy involves using an external magnetic field to control the spin states of catalytic sites and reactants, thereby promoting the spin‐allowed reaction pathway [26, 27]. However, this approach has a major disadvantage as it requires sometimes complex and energy‐intensive equipment to generate a strong and uniform magnetic field, making it impractical for large‐scale industrial applications. Another emerging approach is based on the chiral‐induced spin selectivity (CISS) effect [28, 29]. It is a very promising strategy for manipulating electron spin and enhancing the efficiency of electrochemical reactions.

The CISS effect arises from the interaction between the spin of an electron and its transport through a chiral environment. Chiral molecules or structures, lacking mirror symmetry, can induce a spin polarization of the transmitted electrons, favoring one spin orientation over the other [30]. This phenomenon has significant implications for electron transfer processes, where the spin states of the reactants and products play a crucial role [23, 24, 28, 29, 31, 32, 33, 34, 35, 36, 37, 38]. By utilizing chiral materials as electrocatalysts, it is possible to generate spin‐polarized electrons. For example, Lu et al. demonstrated spin‐selective electron transport through chiral MOFs, underscoring their potential for applications in spintronics and enantioselective catalysis. Other findings further exemplify the versatility of the CISS effect and its relevance for many applications [23, 24, 28, 29, 31, 32, 33, 37, 38, 39]. In particular, it significantly impacts ORR. Naaman et al. explored the influence of chirality on ORR. They found that chiral electrodes, especially those modified with chiral organic layers or composed of chiral nanoparticles, exhibit enhanced ORR performance when compared to their achiral counterparts. This enhancement is attributed to the CISS effect, where chiral structures preferentially transmit electrons with one spin direction. The spin‐polarized electrons generated by the chiral electrodes are believed to facilitate the spin‐forbidden transitions involved in ORR, leading to lower overpotentials and higher current densities [29]. Apart from that, Zhang et al. utilized intrinsically chiral AuNPs as spin filters to couple them with catalytically active surface sites for enhanced ORR and Oxygen evolution reaction (OER) [40]. These findings highlight the possibility of utilizing chiral materials to design more efficient electrocatalysts for fuel cells.

The interaction of oxygen with a surface is a critical step in various chemical processes [41, 42, 43], including catalytic oxidation reactions [44], the formation of oxide films in the semiconductor industry, and corrosion [45]. These processes are influenced by factors such as the kinetics of adsorption, as well as the atomic and electronic structures of the involved surfaces [46, 47, 48]. However, the role of the oxygen's electron spin, arising from its two unpaired electrons, with respect to its adsorption dynamics is not yet fully understood [49, 50]. Kurahashi investigated the effect of molecular alignment and electron spin on the adsorption of O_2_ on various surfaces, including silicon, aluminum, nickel, and tungsten. The study revealed that the sticking probability of O_2_, a measure of how likely it is to adsorb onto a surface, depends strongly on the alignment of the O_2_ molecules and the orientation of their spin relative to the surface [45]. This highlights the importance of spin and steric effects in the adsorption process and plays an important role in the current study, as it allows introducing an additional break of symmetry in terms of spin interactions.

The literature to date has primarily focused on the design of spin‐controlled catalysts or chiral catalysts by assembling chiral molecules, modifying surfaces with chiral organic monolayers, or even with intrinsically chiral materials. However, the influence of chirality, generated in a metal matrix by the imprinting of specific enantiomers, on the CISS effect and the related ORR, remains completely unexplored. Recently, we have successfully developed chiral‐encoded metals with mesoporous features by electrodepositing metals in the simultaneous presence of non‐ionic surfactants and various chiral compounds [51, 52, 53]. These nanostructured designer materials can perfectly retain chiral molecular information after complete template removal. This allows them to be used for various applications, such as the synthesis of chiral compounds, enantioselective analysis, chiral separation, as well as in enantioselective actuators and electrochemiluminescent sensors [52, 53, 54, 55, 56, 57].

In the present contribution, a chiral‐encoded mesoporous platinum‐iridium alloy (Pt‐Ir) is studied as an electrocatalyst for ORR. While the use of noble metals like Pt and Ir provides for this fundamental study a stable platform (e.g., no risk of corrosion) to demonstrate a novel concept, the underlying principles of CISS might also be extended to guide the rational design of more abundant and affordable catalysts. We show that such electrodes can improve ORR efficiency due to the CISS effect when imprinted with the appropriate enantiomer, leading to a significant current enhancement of over 200%. However, in strong contrast with the existing literature, this effect cannot be observed when using an electrode of analog composition, but which has been imprinted with the opposite enantiomer. Most interestingly, in the latter case, oxygen reduction is partially inhibited when compared to an achiral electrode. This intriguing behavior originates from a synergetic coupling of the CISS effect with a preferential spin orientation of adsorbed oxygen and opens up novel perspectives for developing catalysts for ORR.

## 2 Results and Discussion

### 2.1 Characterization and Electrocatalytic Tests of Mesoporous Pt‐Ir

**FIGURE 1:**
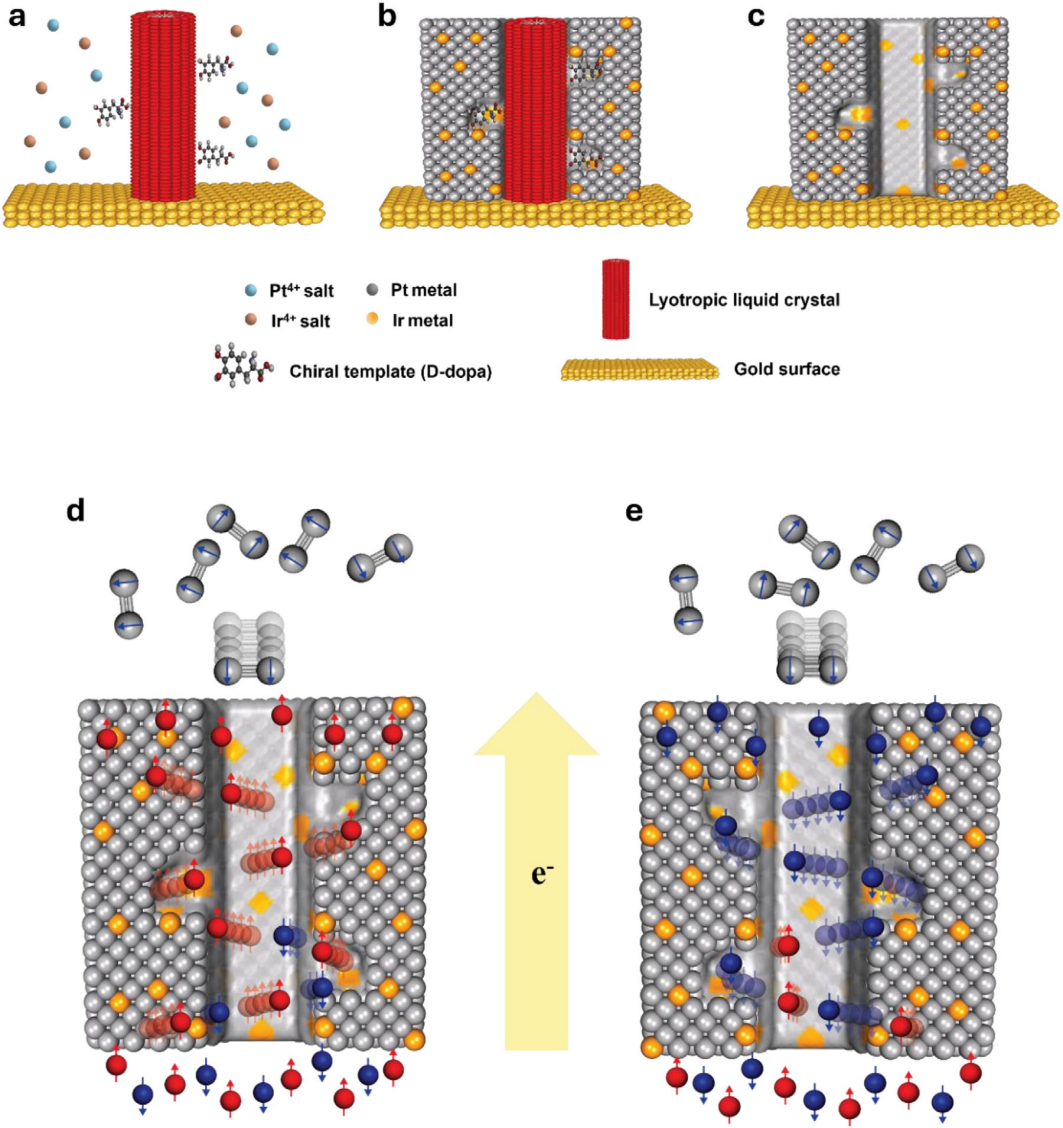
Synthesis of chiral‐encoded mesoporous Pt‐Ir acting as a spin filter. (a) Self‐assembly of a lyotropic liquid crystalline phase with chiral molecules and metal salts (only one surfactant column is shown). (b) Pt‐Ir electrodeposition around the template. (c) Removal of templates, leading to the final chiral‐encoded mesoporous Pt‐Ir film. When oxygen adsorbs at the metal surface during the electrochemical reduction, its spin gets polarized in a preferential direction. This spin alignment is either (d) favorable or (e) unfavorable with respect to the preferential spin orientation of the electrons traveling through the mesoporous metal, depending on the chirality of the imprinted enantiomer.

The synthesis leverages electrodeposition within a lyotropic liquid crystal (LLC) template to generate chiral‐encoded mesoporous Pt‐Ir on a gold‐coated glass support. As shown in Figure 1a–c, the chiral template 3,4‐dihydroxyphenylalanine (DOPA) interacts with the hydrophilic head groups of the self‐assembled LLC phase, containing also a metal salt as a precursor. This interaction influences the spatial arrangement of the LLC structure [52, 58]. Subsequent electrodeposition of Pt‐Ir occurs within this chiral environment, resulting in a chiral‐encoded mesoporous film upon template removal. This approach allows precise control over material growth and the final mesoporous architecture, confirmed by the network of pores observed in subsequent analysis. Figure 1d, e illustrate schematically the chiral‐induced spin selectivity effect of such a material. Figure 1d symbolizes a metal phase propagating preferentially electrons with spin‐up orientation due to the imprinted specific chirality, whereas Figure 1e, imprinted with the opposite chiral information, is more favorable for the transmission of electrons with spin‐down polarization, not matching with the spin orientation of the adsorbed oxygen. This *in fine* leads to either constructive (higher current) or destructive (lower current) interactions with respect to the electron transfer.

To engineer such chiral‐encoded mesoporous Pt‐Ir layers, a previously reported procedure has been used with some modifications [53]. A detailed description of the synthesis of the chiral electrodes, as well as their morphology, electronic structure, XRD, electrochemical properties, and enantioselective recognition abilities, is described in detail in this previous contribution, as well as in the Figures S1–S6 and Table S1.

A first and crucial experiment concerns the chiral character of the elaborated electrodes. This has been tested by exposing an electrode imprinted with D‐DOPA to a solution containing either D‐DOPA or L‐DOPA (Figure S6). In the case of successful imprinting, such an electrode should have a higher affinity for D‐DOPA and consequently should show bigger oxidation currents. Differential Pulse Voltammetry (DPV) has been used to detect the electrochemical oxidation signal of DOPA as a probe molecule. When tested with the two different enantiomers of DOPA, the electrode showed indeed, as expected, a significantly higher signal for D‐DOPA oxidation. This provides direct evidence of the electrode's ability to recognize and distinguish between the two enantiomers of a chiral molecule, and thus its chiral character. We would like to mention that diastereomeric interactions are essential for distinguishing between two enantiomers. Therefore, if different signals are observed for two enantiomers of the same molecule, it necessarily means that the tool which is used to probe the interaction (in our case an electrode) needs to be itself chiral. In addition, these electrodes have been previously used not only for analyzing two enantiomers, but also to synthesize almost enantiopure products (up to 95% enantiomeric excess) starting from a prochiral precursor. This provides another extremely strong evidence for the chiral character of the generated metal layers, because when non‐imprinted electrodes are used for the same synthesis, a 50/50 mixture of both enantiomers is obtained.

Another crucial aspect is to ensure that the chirality of the employed electrodes is not due to chiral template molecules remaining in the metal matrix after the imprinting process. The most direct way to visualize the presence of leftover template molecules is to test the electrodes by DPV in pure supporting electrolyte once they have undergone the overnight washing step. DPV is extremely sensitive to the presence of even tiny amounts of (adsorbed) redox active molecules, and therefore, the absence of signal in the potential window which is characteristic for DOPA oxidation, certifies the absence of chiral molecules in or on the mesoporous metal matrix. In Figure S7, we compare the DPV signal of an imprinted electrode in a blank 50 mm HCl electrolyte (black line) with its response in a standard DOPA solution in the same electrolyte (red line). The complete absence of any characteristic DOPA signal in the blank electrolyte confirms that the DOPA molecules used for imprinting have been entirely removed.

**FIGURE 2:**
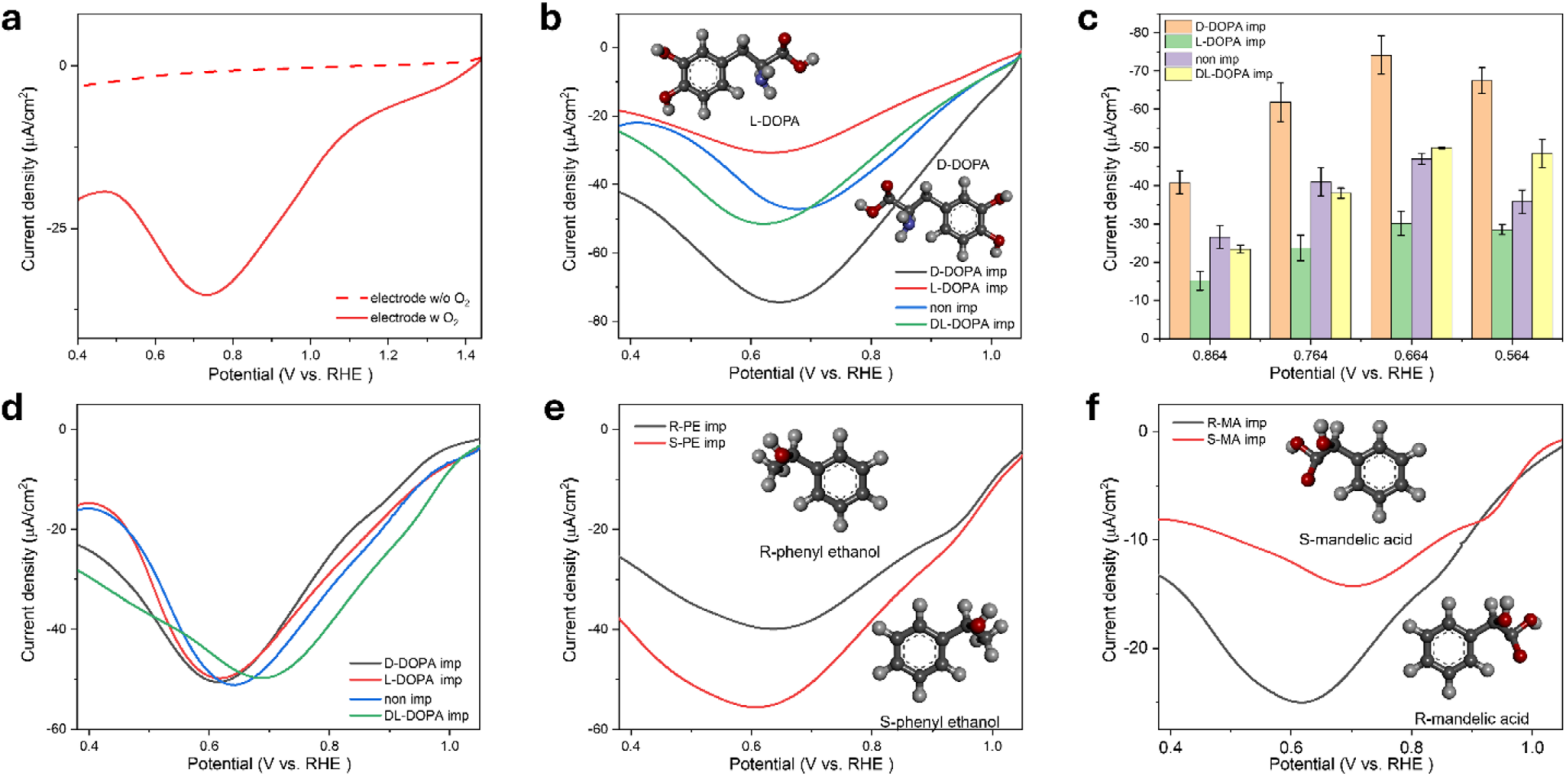
(a) Linear sweep voltammetry (LSV) of a non‐imprinted electrode in the presence of oxygen and without oxygen. (b) LSV of D‐DOPA, L‐DOPA, DL‐DOPA encoded electrodes and a non‐imprinted Pt‐Ir electrode in the presence of oxygen. (c) Current density comparison for the ORR experiment at different potential values, extracted from Figure 2b. (d) LSV of D‐DOPA, L‐DOPA, DL‐DOPA imprinted electrodes, and a non‐imprinted Pt‐Ir electrode after erasing the chiral features by oxidizing the alloy surface. LSV in the presence of oxygen, recorded with mesoporous metals imprinted with different types of chiral molecules (e) R‐ and S‐phenyl ethanol, as well as (f) R‐ and S‐mandelic acid.

To assess the electrocatalytic activity of the chiral‐encoded mesoporous Pt‐Ir electrodes for ORR, Linear Sweep Voltammetry (LSV) has been employed, as shown in Figure 2a. LSV is a technique commonly used to evaluate the current response of an electrode under a gradually changing voltage [59, 60]. Figure 2a presents the LSV curves for a non‐imprinted mesoporous Pt‐Ir electrode in the presence and absence of oxygen. At a scan rate of 10 mV/s, in an oxygen‐saturated solution, a well‐defined peak, stretching from around + 1.0 to + 0.50 V vs. RHE (red solid line), confirms the occurrence of ORR. This peak is absent in a nitrogen‐purged environment (red dashed line), indicating that the observed current increase is solely due to oxygen reduction.

Figure 2b presents the LSV experiments performed to evaluate the influence of chirality on the ORR activity (see Figure S8 for additional scan rates). The chiral features of a fresh mesoporous Pt‐Ir electrode imprinted with D‐DOPA enhance the ORR activity (black curve) compared to the non‐imprinted electrode (blue curve). This is somehow expected, based on the fact that in literature reports dealing with electrodes modified with chiral organic layers, the presence of a chiral surface feature has also been shown to have a beneficial influence on the oxygen reduction rate [29]. In a control experiment, an electrode imprinted with DOPA racemate has also been tested (green curve) and shows current densities very close to those observed for a non‐imprinted mesoporous electrode, which again is not surprising as both electrodes are globally achiral. Analog experiments have also been performed with other chiral template molecules (Figure 2e, f), and these results are discussed further down.

However, what is very unusual is that when employing an electrode imprinted with L‐DOPA (red curve in Figure 2b), the ORR current density decreases with respect to the achiral electrode by an amplitude which is approximately equivalent to the increase that is observed for the D‐enantiomorph. This result is in strong contrast to what is sometimes observed with chiral organic layers, where chirality systematically enhances the oxygen reduction kinetics [29]. These findings are further illustrated in Figure 2c, providing a more quantitative comparison of the current densities measured with each type of electrode during the ORR process at different potential values. Three different electrodes of the same composition (either L‐imprinted, or D‐imprinted, or DL‐imprinted, or non‐imprinted) were measured by LSV like in Figure 2b, and then the current density values were averaged for a selected subset of potential values along these scans, giving the possibility to display a mean value and the associated error bars. The reduction current for the D‐DOPA imprinted electrodes (orange bar) is systematically significantly higher, and the L‐DOPA imprinted electrodes (green bar) exhibit a noticeably lower current density compared to non‐imprinted electrodes (purple) and racemate‐imprinted electrodes (yellow). These different activities are also translated by the measured half‐wave potentials: the D‐DOPA‐imprinted electrode exhibits the most positive half‐wave potential (0.850 V vs. RHE), followed by the non‐imprinted electrode (0.827 V vs. RHE), and the L‐DOPA‐imprinted electrode (0.807 V vs. RHE). This directly supports our claim that D‐DOPA imprinting enhances ORR activity while L‐DOPA imprinting inhibits it. In order to exclude that differences in oxygen mass transport conditions might be at the origin of these observations, these findings were further validated under controlled mass‐transport conditions. LSV was performed using a Rotating Disk Electrode (RDE) at 1600 rpm to eliminate mass‐transport related effects (Figure S9). The results perfectly align with the static electrode measurements: the D‐DOPA imprinted electrode (black curve) exhibits a significantly higher diffusion‐limited plateau current density compared with the L‐DOPA imprinted electrode (red curve). This consistency under dynamic conditions confirms that the observed chirality‐related enhancement and inhibition are intrinsic to the imprinted metal surfaces.

In order to further verify that these current density changes are directly related to the chiral character of the electrodes, we employed a methodology to erase on purpose the chiral features of the electrodes by scanning their potential for 40 cycles from −0.1 to + 1.4 V vs. RHE in sulfuric acid. This process is known to remove the chiral feature by oxidizing the metal surface [53]. As illustrated in Figure S4a, the oxidation of platinum to platinum oxide starts around + 1.0 V vs. RHE, which destroys at least partially the encoded chiral information of the electrode [61, 62]. Subsequently, the oxygen reduction activity of the different electrodes was reevaluated. All electrodes, the initially chiral ones, as well as the control electrodes, exhibit now identical current densities for ORR (Figure 2d). Simultaneously, these electrodes also lose their enantioselectivity with respect to DOPA recognition (Figure S10). The combination of this loss of differentiation in terms of ORR activity and the concomitant loss of the electrodes’ chiral features strongly suggests that the initial enhancement and inhibition observed in Figure 2b is due to the presence of chiral features in the metal matrix. These findings support the crucial role of the CISS effect in promoting the ORR activity of the chiral Pt‐Ir surfaces when they have the appropriate chirality.

To confirm that the observed cathodic current is solely attributable to ORR, without interference from the reduction of platinum oxides (PtO), an additional control experiment was performed (Figure S11). Initially, the fresh Pt‐Ir electrode was tested in an O_2_‐saturated electrolyte, yielding a clear ORR current (Figure S11a). We then performed a subsequent LSV scan with the same, now ‘used’ electrode in an O_2_‐free (N_2_‐purged) electrolyte. As shown in Figure S11b, the characteristic ORR peak vanishes. Upon reintroduction of oxygen into the electrolyte, the ORR peak is immediately restored (Figure S11c). While a slight decrease in the overall current density was observed in this final scan, due to a minor electrode degradation over the extended measurement time, the complete vanishing of the reduction signal in the absence of O_2_ and its subsequent restoration upon re‐saturation provides evidence that the measured cathodic current originates exclusively from ORR. This control experiment confirms that the potential range relevant to ORR is free from interfering oxide reduction processes. It furthermore illustrates that the employed alloy electrodes are sufficiently stable to be used several times, which might also be an advantage compared to the previously reported chiral organic layers that can eventually be more fragile due to gradual desorption.

### 2.2 Variation of Ir Doping and Film Thickness

**FIGURE 3:**
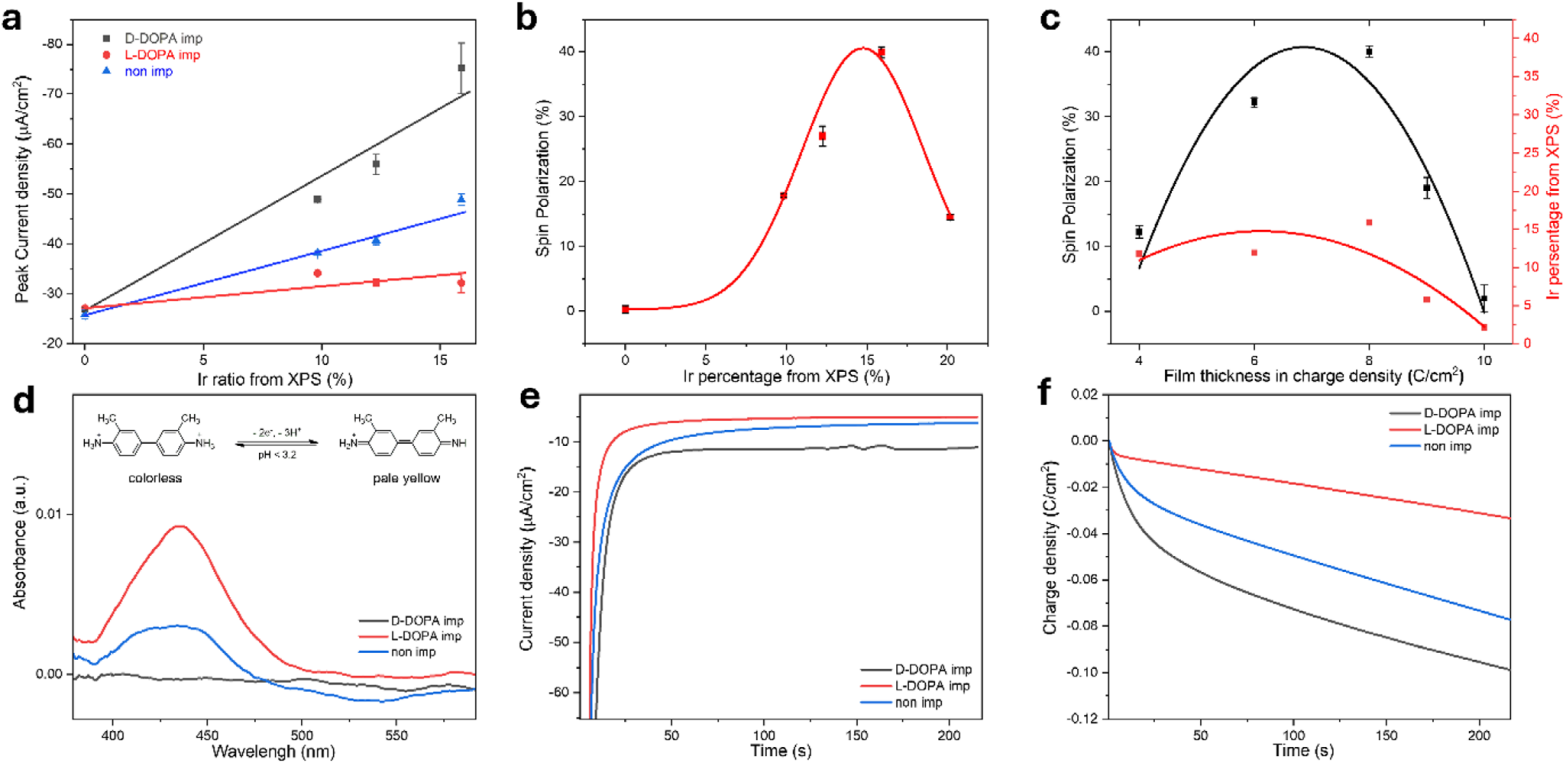
(a) Peak current density comparison for electrodes with different Pt and Ir ratios. (b) Apparent spin polarization percentage for electrodes with varying Pt and Ir ratios (c) Apparent spin polarization percentage and Ir content for different electrodes as a function of the film thickness. (d) UV–vis absorption spectra of o‐tolidine recorded with solutions extracted from the electrochemical cell after chronoamperometry. (e) Chronoamperometry signals recorded at + 0.66 V vs. RHE with D‐DOPA imprinted (black), L‐DOPA imprinted (red), and non‐imprinted electrodes (blue) in oxygen‐saturated solutions (f) Comparison of the global charge density injected for ORR during the chronoamperometry experiments of (e).

To gain a deeper understanding of the factors influencing the observed electrocatalytic activity, we investigated the impact of different Pt‐Ir ratios and film thicknesses on the electrode performance. Varying these two parameters should provide a better insight into their role in optimizing the CISS effect. Figure 3a illustrates the impact of the degree of Ir doping on the peak current density for D‐DOPA (black squares) and L‐DOPA (red circles) imprinted electrodes. The peak current density, a measure of the maximum ORR rate, strongly depends on the amount of Ir and the type of chiral imprint. The Ir ratios reported in the graph are derived from the XPS measurements detailed in Table S1. For the D‐DOPA imprinted electrodes, the peak current density increases with the Ir content up to around 15% of Ir (Figure S12 shows a subset of LSV curves used for the peak current density calculations), suggesting a synergistic effect between Pt and Ir in promoting ORR. In strong contrast, the peak current density of the L‐DOPA imprinted electrodes remains relatively low for all Pt‐Ir ratios. It is also systematically lower than the one recorded for non‐imprinted electrodes. The difference in peak current densities between D‐DOPA and L‐DOPA imprinted electrodes allows calculating the apparent values of the spin polarization (Equation S1) induced by the chiral imprint. This simple ratio does not directly indicate the real spin‐polarization, but it is a measure of the asymmetry between signals, which in the CISS community is often used for describing spin‐polarization. In principle, this measure of asymmetry provides directly the spin‐polarization only for photo‐spectroscopy using Mott scattering, but not for other types of measurements. However, several publications employ it as a means to quantify in a first‐order approximation the spin polarization [63], also for electrochemical current measurements [64]. Therefore, this term of apparent spin polarization should be just considered as an analogy to the real physical definition of spin polarization, but is most likely the best way to quantify the asymmetry of the system, which is related to the chiral character of the metal layers. All other experimental parameters, such as mass transport, morphology, surface area, and active site distribution, intrinsically cannot be different when just switching from one enantiomer to its mirror image, as they have by definition identical chemical and physical features. Figure 3b quantifies this apparent spin polarization as a function of the Ir percentage, and it is important to note that when there is no Ir present in the metal layer, there is no observable difference in peak current density between the two enantiomorphs, indicating no spin polarization. However, as the Ir content increases, spin polarization increases with the Ir content, suggesting that the presence of Ir plays a crucial role in the CISS effect, most likely originating from spin‐orbit coupling. However, beyond 15% Ir, the apparent spin polarization decreases, indicating an upper limit for the beneficial impact of Ir. Figure 3c illustrates the relationship between the apparent spin polarization, the actual Ir content of the electrode, and the film thickness, directly correlated with the charge density (C/cm^2^) that was used during the electrodeposition step (see also Figure S4). In this experiment, the initial ratio of Pt and Ir in the plating gel was 85:15. The deduced apparent spin polarization percentage initially increases with film thickness, reaching a maximum at around 8 C/cm^2^, which approximately corresponds to a layer thickness of 1.2 µm, with little difference in Ir content. This initial rise can be attributed to the fact that a thicker film can act as a better spin filter, thus enhancing the CISS effect. Beyond 8 C/cm^2^, the spin filtering effect decreases. This decline can be correlated with a decreasing Ir content at the outermost film surface when the thickness increases, which is due to a relative depletion of Ir precursor compared to Pt precursor in the plating gel for longer electrodeposition times. This can be explained by the fact that the Ir precursor is present only in rather low concentrations in the plating gel, and therefore it gets gradually depleted during the electrodeposition [53]. Consequently, this gradient of Ir percentage is also responsible for the decrease of the spin polarization analog, which was observed for thicker alloy layers (see Figure 3b, c). To summarize, Ir plays a major role in enhancing the CISS effect, and its lower concentration at the surface of thicker electrodes diminishes the overall apparent spin polarization because, before reaching the electrode/electrolyte interface, the electrons will travel through this less spin‐selective part of the layer and partially lose their polarization.

### 2.3 Study of Hydrogen Peroxide Generation

ORR can proceed through two distinct pathways, either via a four‐electron process or by a transfer of just two electrons, the latter one leading to hydrogen peroxide (H_2_O_2_) as a product [65, 66], which, at the basic pH conditions of our experiments, is actually present as HO_2_^−^. To investigate the selectivity of the chiral‐encoded electrodes with respect to these pathways, two experiments were carried out to detect the amount of produced HO_2_^−^. In the first experiment, we employed the oxidation reaction of Fe^2^^+^ to Fe^3^^+^, which is known to be promoted by the presence of hydrogen peroxide (Figure S13). The results indicate that the D‐DOPA imprinted electrode produces less HO_2_^−^ during ORR, suggesting a higher selectivity in favor of the four‐electron pathway, directly reducing oxygen to water. In the second experiment, we employed o‐tolidine as an indicator for HO_2_^−^. o‐tolidine reacts with hydrogen peroxide to form a colored product (see Figure 3d), and the color intensity, measured at 430 nm, is proportional to the produced HO_2_^−^ concentration. The solution obtained with the D‐DOPA imprinted electrode exhibits the lowest absorbance, indicating the lowest HO_2_^−^ formation, while the L‐DOPA imprinted electrode produces a solution that shows the highest absorbance, related to the highest HO_2_^−^ production. The non‐imprinted electrode falls in between. These results collectively demonstrate that the D‐DOPA imprinted electrode, which exhibits the highest efficiency for ORR, also has the highest selectivity toward the four‐electron pathway, minimizing the undesirable production of HO_2_^−^. On the contrary, the L‐DOPA imprinted electrode, which shows the lowest ORR kinetics, is more favorable for the two‐electron path, leading to increased HO_2_^−^ formation. The non‐imprinted electrode exhibits an intermediate behavior.

To further confirm the impact of chirality on spin‐dependent processes, we investigated the electrochemical behavior of D‐DOPA and L‐DOPA imprinted electrodes in a solution containing K_3_[Fe(CN)_6_], a redox couple known to involve only a one‐electron transfer process [67]. As shown in Figure S14, the CVs of both electrodes exhibit nearly identical Fe^2+^/Fe^3+^ oxidation‐reduction peaks. This observation indicates that the chiral character of the imprinted electrodes does not influence a one‐electron transfer process, suggesting that the CISS effect is only relevant for reactions which are sensitive to spin‐polarized electrons, such as ORR. Figure 3e illustrates the chronoamperometry results, showcasing the current response for D‐DOPA imprinted, L‐DOPA imprinted, and non‐imprinted electrodes at a constant potential of + 0.66 V vs. RHE in oxygen‐saturated solutions. The D‐DOPA imprinted electrode consistently exhibits a higher current density than the L‐DOPA imprinted and non‐imprinted electrodes. Integrating the area under the chronoamperometry curves allows quantifying the overall charge used during ORR for each electrode. Figure 3f compares the transferred charge densities for the D‐DOPA imprinted, L‐DOPA imprinted, and non‐imprinted electrodes. The D‐DOPA imprinted electrode exhibits a significantly higher overall charge density, which allows calculating again the apparent spin polarization. The obtained value of 44% is in good agreement with the spin selectivity obtained by LSV and further supports the significantly enhanced ORR activity, translated into an up to 242 ± 45% increase in current or charge for the D‐DOPA‐encoded electrode compared to its antipode (see Supporting Information). Since OH^−^ (or HO_2_^−^) is preferentially formed with higher (or lower) concentrations from ORR when the provided electrons are spin‐polarized with the right orientation, while the opposite appears to be true when they are not, the suggestion is that OH^−^ is preferentially produced when a triplet‐like pair of electrons is provided to O_2_. On the contrary, HO_2_^−^ production dominates when the electrons are provided with the wrong spin orientation. In the first scenario (matching triplet pairs), the spin‐angular momentum is conserved by combining two triplets with opposite *m_z_*, whereas in the second scenario, electron transfer occurs between singlet states.

### 2.4 Oxygen Adsorption for Breaking the Symmetry

**FIGURE 4:**
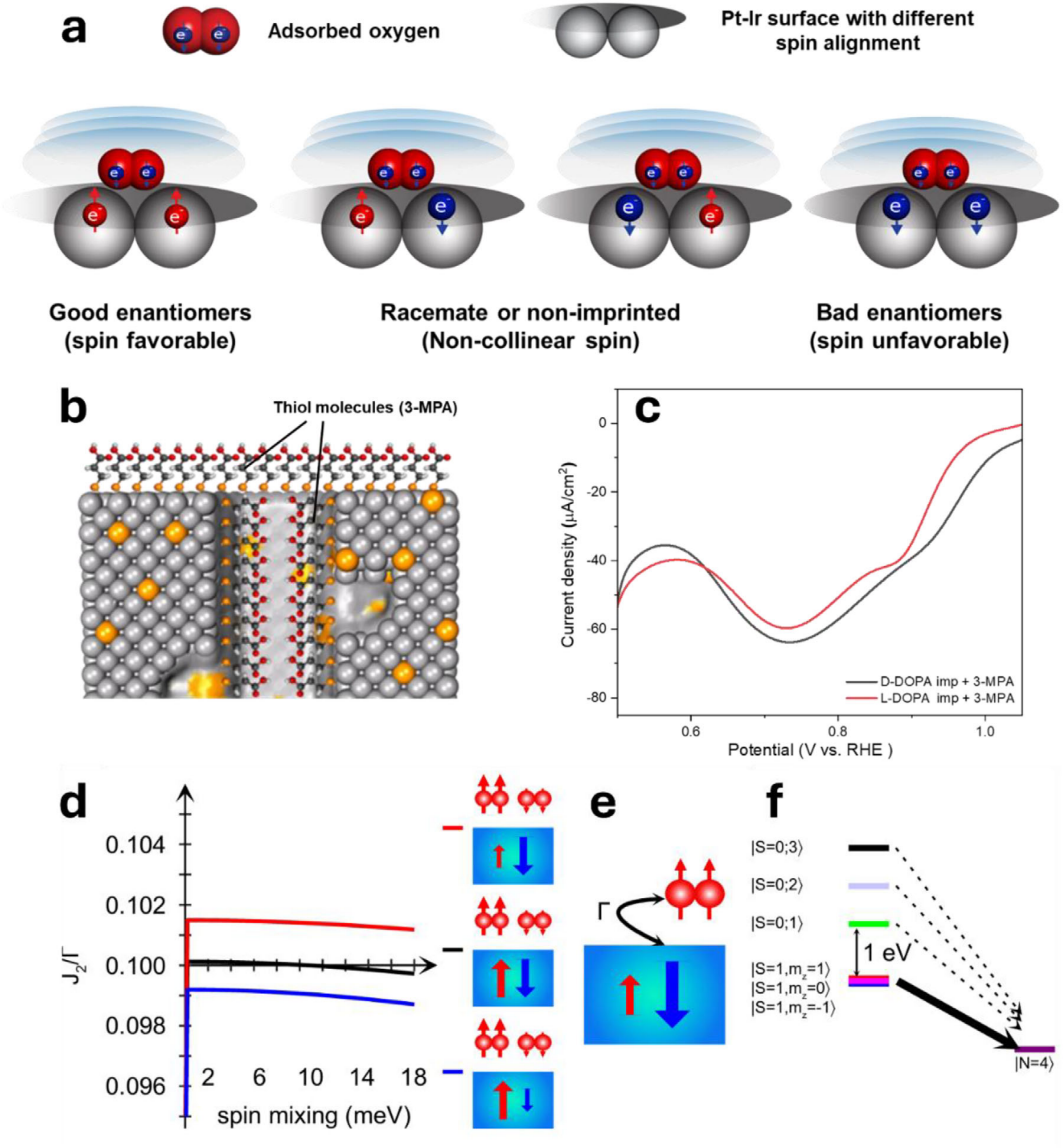
(a) Schematic illustration of electrons traveling through the metal layer (symbolized by the two gray metal atoms), interacting with the electrons of the oxygen molecule (in red) adsorbed at the electrode/electrolyte interface (indicated by the gray disc). Depending on their relative spin orientations, the interaction is favorable (left), neutral (middle), or unfavorable (right), leading to higher, intermediate, or lower current densities, respectively. (b) Schematic illustration of the blocking of direct oxygen adsorption on the metal surface via the grafting of a self‐assembled monolayer (SAM) of 3‐mercaptopropionic acid (3‐MPA) on the mesoporous structure. (c) Linear sweep voltammetry measurements of two SAM‐modified electrodes having opposite chirality in an oxygen‐saturated solution. (d) Simulated results of the current corresponding to the two‐electron transfer (J_2_) as a function of the spin‐mixing. The current is scaled by the coupling energy (Γ) between the O_2_‐molecule and the Pt surface. The three curves represent the transfer currents for unpolarized injected electrons (black line), and spin‐polarized electrons with a majority spin down (red line) and spin up (blue line). The spin mixing corresponds to the degree of spin‐orbit coupling induced by the Pt surface on the O_2_‐molecule. (e) Schematic of the O_2_‐molecule adsorbed on the metal with a coupling energy Γ. (f) Energy diagram of the direct two‐electron transfer between the two‐ and four‐electron states of the O_2_‐molecule.

The above‐described results, clearly indicating that one enantiomorph accelerates the electron transfer, whereas its mirror‐symmetric counterpart leads to a partial inhibition of the four‐electron transfer, need to be understood from a mechanistic point of view. In the so far existing reports, the presence of chiral features on the electrode surface usually catalyzes ORR, no matter the type of chirality (left‐ or right‐handed) [23, 24, 28, 29, 31, 32, 33, 34, 35]. This has been convincingly explained by the fact that, due to the spin filtering effect of the chiral layer, the spins of the majority of the transmitted electrons arriving at the electrode/electrolyte interface are aligned in the same direction and therefore can advantageously match with the spins of triplet oxygen. Indeed, in this case, the absolute chiral configuration of the surface layer does not matter, as long as the electrons are transmitted with a coherent parallel spin alignment, because the oxygen molecule, arriving from the bulk solution by diffusion, has no preferential orientation and also no specific spin alignment with respect to the surface. The latter point is only valid as long as one considers that the oxygen molecules have no particular interaction with the surface, which is a reasonable assumption for surfaces modified with chiral (bio)organic layers. However, in the present work, due to the absence of organic layers, oxygen is able to interact strongly with the bare metal surface, exposed to the solution [68]. Such an interaction between the metal surface and oxygen has an impact on its spin state [69], and it is reasonable to assume that it leads to a certain degree of spin polarization due to spin‐orbit coupling [70, 71]. Spin‐state‐resolved studies of O_2_ sticking on surfaces have shown that the O_2_ sticking probability depends strongly on the molecular alignment and the spin orientation of O_2_ relative to the surface [45]. Thus, it is more likely to find at the surface oxygen molecules with a certain preferential spin orientation compared to the opposite one. Consequently, one can assume that the adsorbed oxygen acquires a dominating spin orientation, which either matches or not the orientation of the spins of the electrons that are transmitted through the chiral mesoporous metal layer, as schematically illustrated in Figure 4a. Therefore, this strong interaction of the oxygen molecules with the surface is the essential ingredient to explain the breaking of symmetry in the present system. Using an electrode with one chirality will enhance the electron transfer due to a favorable spin alignment of the electrons traveling through the chiral layer with the pre‐oriented spins of the adsorbed oxygen, whereas the electrode having the opposite chirality will significantly inhibit the electron transfer due to a violation of spin‐angular momentum conservation. For achiral electrodes, either non‐imprinted or imprinted with racemate, the situation is neither favorable nor unfavorable, and consequently, the measured currents lie between the previous two cases.

If this hypothesis is correct, it should be possible to decrease or suppress the discriminating effect by eliminating the direct interaction of oxygen with the metal surface. We verified this by modifying the entire mesoporous Pt‐Ir matrix with a monolayer of 3‐mercaptopropionic acid (3‐MPA). The short self‐assembled monolayer, generated by dipping the electrodes for 2 h in a 10 mm solution of 3‐MPA in ethanol, covers not only the outer surface of the metal layer, but also the inside of the mesoporous structure. Thus, oxygen can no longer adsorb (Figure 4b), and the D‐DOPA and L‐DOPA imprinted electrodes exhibit very similar ORR currents (Figure 4c). Interestingly, after this monolayer modification, the current density of the L‐DOPA‐imprinted electrode increases, whereas the D‐DOPA‐imprinted electrode delivers lower currents when compared to their analogs without SAM. This indicates that the adsorption of oxygen on the surface of the chiral electrodes is the essential ingredient for breaking the symmetry of the overall system and for explaining the opposite impact of both enantiomorphs on ORR. An additional possibility to confirm these findings is to study the associated hydrogen peroxide formation. When blocking the surface of two electrodes, one imprinted with D‐DOPA and the other with L‐DOPA, with a SAM, both electrodes also show the same amount of hydrogen peroxide production (Figure S15). This suggests that, due to the absence of oxygen adsorption, the type of preferential spin orientation of the electrons transmitted through the metal matrix no longer has an impact on the reaction pathway (2e or 4e ORR). This again indicates the importance of oxygen adsorption for the breaking of symmetry in the system, leading to the observed dual inhibition‐catalysis effect.

### 2.5 Calculation of Spin‐metal‐oxygen Interactions

In order to better understand the details of the possible mechanism leading to the observed differences between the two enantiomorphic electrodes, we performed calculations of the electron transfer between the metal substrate and the oxygen molecule.

To this end, we investigated the effect of supplying spin‐polarized electrons to O_2_ molecules. The modeling of this aspect was performed according to the approach outlined in previous publications [72, 73], where the O_2_ valence is modeled as a spin dimer with a triplet‐singlet splitting of about 1 eV. In the calculation, we address the absorption of two additional electrons in the spin dimer, thereby monitoring transitions between the two‐ and four‐electron states, either directly or via the three‐electron route. The former type of process corresponds to a spin angular momentum conserving two‐electron transfer process, whereas the latter to single‐electron transfer, which is enabled whenever there is a non‐negligible spin‐orbit coupling in the system.

Specifically, we wanted to test whether a predefined spin‐orientation of the O_2_ molecule, when adsorbed on the metal surface, is sensitive to a spin‐polarized current. This question is more relevant in systems with strong spin‐orbit coupling, partly since it may destroy the spin‐polarization and partly because it may hamper the correlated two‐electron absorption. Our calculations show that a spin‐polarized current either increases or decreases the electron transfer rate with respect to the rate defined for a non‐spin‐polarized current, corroborating our hypothesis outlined in Figure 4a.

The results from these simulations are shown in Figure 4d–f, where the two‐electron transfer current J_2_ is plotted as a function of spin‐mixing. The current is scaled by the coupling energy between the O_2_‐molecule and the Pt surface, and the spin‐mixing corresponds to the degree of spin‐orbit coupling induced by the Pt surface on the O_2_‐molecule. The plot shows the transfer current for spin‐polarized (red, blue) and unpolarized (black) injected electrons, under the assumption that O_2_ is more likely to adsorb on the Pt surface in the spin‐triplet configuration with m_z_ = 1 than with m_z_ = −1. For injected electrons with majority spin down (red line), there is an increased probability for a matching angular momentum between the injected electrons and the adsorbed O_2_‐molecule compared to the unpolarized electrons (black line). Analogously, for the majority spin‐up configuration (blue line), matching angular momentum is less probable.

### 2.6 Activity of Electrodes Imprinted with Other Chiral Molecules

The versatility of the proposed concept can be demonstrated by elaborating electrodes imprinted with other types of chiral molecules besides DOPA. Figure 2e, f presents the LSV curves in the presence of oxygen for electrodes imprinted with R‐ and S‐phenylethanol, as well as R‐ and S‐mandelic acid, respectively. A clear difference in ORR activity is again observed for electrodes imprinted with opposite enantiomers in both cases. The S‐phenylethanol imprinted electrode exhibits a higher current density compared to the R‐phenylethanol analog (Figure 2e). Similarly, the R‐mandelic acid imprinted electrode outperforms the S‐mandelic acid imprinted version (Figure 2f). Both of the current‐enhancing enantiomers have the same stereochemistry as D‐DOPA, despite the opposite assignment of R and S due to nomenclature rules. Notably, the enantiomers exhibiting lower catalytic activity display a shoulder peak at approximately 0.9 V vs. RHE. This feature corresponds to the 2‐electron reduction process, suggesting a change in reaction mechanism. These observations support the hypothesis that the less active electrode directs the ORR toward a 2‐electron pathway, in contrast to the more active enantiomorph, which favors the 4‐electron pathway. These results indicate that the concept of utilizing chiral‐imprinted metals to enhance ORR activity is not limited to DOPA as a molecular template, but can be extended to other chiral molecules. The consistent observation of enhanced ORR activity for one enantiomorph compared to its antipode reaffirms the role of chirality in promoting efficient oxygen reduction.

## 3 Conclusions

This study demonstrates the presence of a chiral‐induced spin selectivity (CISS) effect when using chiral‐encoded Pt‐Ir surface layers for the oxygen reduction reaction (ORR). We have shown that when a given enantiomer of chiral molecules, such as D‐DOPA, S‐phenylethanol, and R‐mandelic acid, is used as a templating agent during the electrodeposition of mesoporous Pt‐Ir layers, the ORR activity of the generated electrodes is significantly enhanced compared to an achiral analog and proceeds preferentially via a four‐electron pathway, thus avoiding the production of hydrogen peroxide. This enhancement is attributed to a synergy between the CISS effect and the surface‐adsorbed state of the oxygen molecules, leading to a matching alignment of electron spins. In addition, and most importantly, when the corresponding stereochemical antipodes are used as templates, currents are significantly smaller compared to the achiral electrode configurations. Thus, the same type of electrode material can act either as an activator or as an inhibitor for ORR, depending on the chirality of the imprinted enantiomer. As the two enantiomers of the same chiral molecule have by definition exactly the same physical and chemical properties, it is excluded that imprinting with the two different enantiomers of the same chiral molecule results in different surface morphologies, surface areas, or crystallographic facets that might account for the differences in catalytic activity that were observed. Any morphological changes induced by the imprinting process would be exactly identical for both enantiomers, making it an insufficient explanation for the opposing catalytic effects (enhancement vs. inhibition). The observed opposite trends are strongly related to the fact that during its reduction, oxygen goes through an adsorption step, potentially inducing a preferential spin orientation, which either matches or not with the spin polarization of the electrons traveling through the metal phase, which in this case acts as a spin filter matrix. If the adsorption of oxygen is suppressed by a self‐assembled monolayer of a short molecule having a thiol function, both types of electrodes with opposite chirality show the same ORR activity. Therefore, the present contribution suggests that the CISS effect does not only occur when electrons are traveling through chiral organic molecules (like amino acids, proteins, DNA, helicenes, etc.), as used in many previous studies, but also through a chiral matrix with a completely different composition and structure. This considerably broadens the range of potential applications of this concept. As the chiral character in the present case is a bulk feature of the metal matrix, it is also most likely more stable than an organic monolayer, which might be quite easily removed either by reduction or oxidation.

The thickness of the mesoporous layer, as well as its Ir content, has a strong influence on the spin filter efficiency of the Pt‐Ir matrix, related to a variation in spin‐orbit coupling intensity. The ORR activity increases and decreases for the two opposite enantiomorphs with an increasing Ir content up to a maximum around 15% Ir. This is due to the fact that doping with Ir can enhance the broken symmetry of the electronic properties of the matrix, which amplifies the CISS effect. However, beyond this value, the ORR activity of both enantiomorphs is again quite similar. Apparent spin polarization values of up to 40%, based on the macroscopic electrocatalytic measurements, are observed under optimized conditions, which translates into an over 200% increase in ORR current, when the metal matrix is imprinted with the appropriate enantiomer, for otherwise identical electrode materials. Obviously, the primary goal of the work is not to demonstrate a new “best‐in‐class” catalyst for ORR to beat any records, but to provide a proof of principle that for one and the same electrode matrix, its intrinsic chiral features have a tremendous effect on its activity. Future studies, such as direct measurements of the spin alignment via spin‐polarized photoemission, should allow for further optimization of these systems. Also, exploring a broader range of more affordable and abundant metals or alloys will enable an extension of the use of these chiral encoded metal surfaces for other multi‐electron transfer reactions. Thus, this approach can potentially improve the performance of fuel cells and other electrochemical devices. Beyond the primary scope of electrocatalysis, the observed break of symmetry, due to the adsorption of oxygen, might also have some consequences with respect to possible explanations of the emergence of a preferential chirality in biological systems.

## Conflicts of Interest

The authors declare no conflicts of interest.

## Supporting information




**Supporting File 1**: advs75175‐sup‐0001‐SuppMat.docx.

**Supporting File 2**: advs75175‐sup‐0002‐SuppMat.docx.

## Data Availability

The data that support the findings of this study are available from the corresponding author upon reasonable request.
