# Supplementary material for: Chiral‐Encoded Pt‐Ir Surfaces as Apparent Spin Filter for Enhanced Oxygen Reduction
Source: Adv Sci (Weinh). 2026 Apr 10;13(36):e75175. doi: 10.1002/advs.75175 (PMC13317554; doi:10.1002/advs.75175)
Supplement: Supplementary file 1 — Supporting File 1: advs75175‐sup‐0001‐SuppMat.docx. [file ADVS-13-e75175-s002.docx]

**Supporting Information**

**Chiral-Encoded Pt-Ir Surfaces as Apparent Spin Filter for Enhanced Oxygen Reduction**

Zikkawas Pasom,^[a],[b]^ , Krissanapat Yomthong^[a]^, Sopon Butcha^[a],[b]^, Jonas Fransson*^[c]^, Chularat Wattanakit*^[a]^, and Alexander Kuhn*^[a],[b]^

[a] School of Energy Science and Engineering, Vidyasirimedhi Institute of Science and Technology, 21210 Rayong, Thailand.

[b] University of Bordeaux, CNRS, Bordeaux INP, ISM UMR 5255, 33607 Pessac, France.

[c] Department of Physics and Astronomy, Uppsala University, Box 516, 751 21 Uppsala, Sweden.

E-mail: [jonas.fransson@physics.uu.se](mailto:jonas.fransson@physics.uu.se); [chularat.w@vistec.ac.th](mailto:chularat.w@vistec.ac.th); [kuhn@enscbp.fr](mailto:kuhn@enscbp.fr)

**Table of Contents**

| Index | Caption | Page |
| --- | --- | --- |
| Figure S1 | Top-view SEM images of a mesoporous Pt-Ir electrode at different magnifications a) scale bar 100 µm and b) scale bar 1 µm. These SEM images reveal a smooth surface morphology, indicating a successful and homogenous electrodeposition process of a mesoporous Pt-Ir layer | S6 |
| Figure S2 | a) HR-TEM image of a Pt-Ir film (scale bar 10 nm) and b) TEM image of a mesoporous Pt-Ir electrode. The Pt-Ir film contains lattice fringes, with d-spacing values of 0.24 nm and 0.20 nm, matching with the (111) and (200) planes, respectively, being intermediate between the characteristic plane spacing of pure platinum and iridium, confirming the presence of the two metals in the mesoporous structure. | S6 |
| Figure S3 | SEM images of the cross-section of electrodes with different thickness, generated by an injected charge density of (a) 4 C/cm^2^ and (b) 10 C/cm^2^ during metal deposition (scale bar 1 µm). | S7 |
| Figure S4 | a) Cyclic voltammetry (CV) curves of electrodes with varying thickness in 0.5M H_2_SO_4_ solutions and b) plot of a comparison between the layer thickness, the electrochemically active surface area (ECSA) and the injected charge density during electrodeposition. | S7 |
| Figure S5 | XPS signal for a) platinum and b) iridium, of alloy electrodes with varying thicknesses (4-10 C/cm^2^). XPS results show that standard monometallic Pt exhibits Pt 4*f*_7/2_ and Pt 4*f*_5/2_ peaks at 70.9 and 74.3 eV, respectively, while standard monometallic Ir shows Ir 4*f*_7/2_ and Ir 4*f*_5/2_ peaks at 60.8 and 63.7 eV (yellow vertical lines). Notably, the characteristic doublets of both Pt and Ir in the Pt-Ir films are shifted to higher binding energies. While the Pt peak shifts were consistent across all electrodes, the Ir peaks exhibited a more pronounced shift towards higher binding energies for lower deposition charge densities (higher relative Ir content). | S8 |
| Figure S6 | Differential pulse voltammograms (DPVs) of the electrooxidation of D-DOPA (black) and L-DOPA (red) recorded with D-DOPA imprinted Pt-Ir electrodes to verify the successful transfer of chirality to the Pt-Ir electrode. | S9 |
| Figure S7 | Differential Pulse Voltammograms (DPVs) of a fresh DOPA imprinted electrode, after overnight washing, in blank electrolyte of 50 mM HCl (black line) and in a 4 mM DOPA solution in 50 mM HCl (red line). | S9 |
| Figure S8 | Linear sweep voltammograms (LSV) of D-DOPA and L-DOPA imprinted electrodes in oxygen-saturated solutions with varying scan rates a) 50mV/s, b) 10 mV/s, c) 5 mV/s and d) comparison of LSVs of D-DOPA imprinted electrodes at different scan rates. | S10 |
| Figure S9 | Linear sweep voltammograms (LSV) of D-DOPA and L-DOPA imprinted electrodes in oxygen-saturated solutions using a rotating disk electrode with rotating speed at 1600 rpm. The experiments are carried out under otherwise identical conditions as with the static electrodes. | S10 |
| Figure S10 | Differential pulse voltammograms (DPVs) of the electrooxidation of D-DOPA (black) and L-DOPA (red) recorded with an initially D-DOPA imprinted Pt-Ir electrode for which the chiral character has been destroyed by scanning the potential for 40 cycles from -0.1 V to +1.4 V vs RHE in sulfuric acid. | S11 |
| Figure S11 | a) Electrochemical test of a D-DOPA imprinted mesoporous electrode of a) fresh electrode in O_2_ saturated solution, b) already used electrode in N_2_ saturated solution, and c) already used electrode in O_2_ saturated solution. | S11 |
| Figure S12 | Linear sweep voltammograms (LSV) of D-DOPA imprinted electrodes at different percentage of Ir doping. | S12 |
| Figure S13  Figure S14  Figure S15  Figure S16 | UV-Vis absorption spectra of a Fe^3+^ containing solution extracted from the electrochemical cell after chronoamperometric reduction of an oxygen-saturated solution at a potential of +0.66 V vs RHE, revealing a significantly lower concentration of Fe³⁺ when a D-DOPA imprinted electrode is used as a working electrode compared to L-DOPA imprinted or non-imprinted ones.  Cyclic voltammetry of D-DOPA and L-DOPA-imprinted electrodes in 50 mM K_3_[Fe(CN)_6_] and 0.5 M H_2_SO_4_.  UV-Vis absorption spectra of a Fe^3+^ containing solution extracted from the electrochemical cell after chronoamperometric reduction of an oxygen-saturated solution at a potential of +0.66 V vs RHE, with a D-DOPA and a L-DOPA imprinted electrode, but both have been modified with a self-assembled monolayer of cysteine. This prevents the adsorption of oxygen and therefore the type of spin orientation transmitted through the chiral matrix has no longer an impact on the reaction pathway (2e or 2e oxygen reduction). Consequently the same amount of hydrogen peroxide is produced with both electrodes.  Schematic representation of the steps for the synthesis of a chiral imprinted mesoporous Pt-Ir electrode. a) Mixing of the precursors. b) Electrodeposition process. c) Final electrode after washing out the molecular templates. | S12  S13  S13  S14 |
| Table S1 | Nominal platinum-iridium ratios based on precursor masses compared to actual ratios determined by XPS measurements Quantitative analysis revealed a decrease in iridium content with increasing charge density/film thickness, attributed to the limited Ir precursor concentration in the plating gel. | S14 |
|  |  |  |

**Supplementary Experimental Procedures**

**Characterization**

Scanning electron microscopy (SEM) was employed using a JEOL JSM-7610F instrument to record both, top-down and cross-sectional views of samples prepared with different charge densities to investigate the surface topography and thickness of the chiral-encoded films. Furthermore, the mesoporous architecture of the encoded Pt-Ir alloy was verified via transmission electron microscopy (TEM) using a JEOL JEM- ARM200F microscope operating at 200 kV to ensure a clear visualization of the mesoporous structure. The samples were prepared by carefully detaching the mesoporous Pt-Ir alloy film from the underlying gold-coated glass slide. The detached film was then directly placed onto a carbon-coated copper TEM grid. X-ray photoelectron spectroscopy (XPS) analysis was carried out on a JEOL JPS-9010 instrument equipped with a monochromatic AlKα X-ray source. A low-energy electron flood gun was utilized for sample pretreatment. The carbon 1s (C 1s) peak at 284.7 eV served as the reference for charge correction of the spectra. The mesoporous Pt-Ir film was etched using an argon ion gun for 160 seconds to obtain XPS depth profiles. The etching rate was estimated to be approximately 2.67 nm/s. Based on this etching rate, the total thickness of the Pt-Ir layer removed after 160 seconds of etching is around 430 nm. Electrochemical characterization of all electrodes was performed with an Autolab PGSTAT204 potentiostat, utilizing a three-electrode setup. This setup consisted of an Ag/AgCl (saturated KCl) reference electrode, a platinum mesh counter electrode, and the prepared Pt-Ir alloy electrodes serving as the working electrode. Despite the fact that an Ag/AgCl reference electrode might undergo slow degradation due to AgO formation in strong alkaline electrolytes, it has been chosen as a convenient reference because the formation of AgO is minimal for a 0.1 M KOH solution and does not significantly impact its stability over the short duration of the ORR performance tests (<min). The electrochemical surface area (ECSA) of the chiral-encoded mesoporous Pt-Ir alloy was determined by calculating the integrated area of its hydrogen adsorption peaks, obtained by cyclic voltammetry in 0.5 M H_2_SO_4_ at a scan rate of 10 mV/s between -0.3 and 1.2 V vs Ag/AgCl, and compared to those of a planar Pt-Ir alloy electrode.

**Electrochemical chiral recognition**

The study of the chiral discrimination ability was carried out with L- or D-DOPA imprinted mesoporous Pt-Ir electrodes, elaborated with a deposition charge density of 8 C cm⁻². Differential pulse voltammetry (DPV) was employed for the analysis, with the following parameters: step potential of 10 mV, modulation amplitude of 50 mV, modulation time of 50 ms, and interval time of 500 ms. The electrolyte consisted of a 50 mM HCl solution containing 4 mM DOPA.

**Electrocatalytic activity**

The experiments were carried out with a three-electrode setup in a closed system. A standard reference electrode (Ag/AgCl) and a platinum wire served as the reference and counter electrodes, respectively. The working electrode, with a fixed geometric area of 0.25 cm^2^, was positioned at the cell bottom and remained stationary throughout the measurements. An electrochemical workstation (Autolab PGSTAT204) controlled the measurements conducted at room temperature in a 0.1 M KOH solution. The solution was purged with oxygen for 30 minutes before each test to ensure a consistent and reproducible oxygen level. The cell was then sealed with openings only for the electrodes and an oxygen-purging needle. This needle was removed during measurements to maintain a stable current, but remained above the solution to prevent oxygen depletion. Linear sweep voltammetry (LSV) has been used and the potential scans were conducted by scanning the potential cathodically (from high potentials toward lower potentials). The recorded signals were not corrected for ohmic drop as one can reasonably assume that iR drops are negligible for a 0.1M KOH solution. Furthermore, as the composition of the electrolyte doesn’t change in terms of conductivity when one enantiomer is used or the other, an eventually existing significant ohmic drop would be exactly the same for all experiments.

The apparent spin polarization percentage was calculated by using Eq (1)

polarization percentage =$\text{ }\left[ \frac{\text{j}_{\text{D}}\text{-}\text{j}_{\text{L}}}{\text{j}_{\text{D}}\text{+}\text{j}_{\text{L}}} \right]$ ×100 (1)

with j_D_ and j_L_ being the peak current intensities of D-imprinted and L-imprinted electrodes, respectively. The standard error of the mean of the polarization percentage is calculated by Eq (2)

s.e.m.=$\text{ }\frac{\text{S}}{\sqrt{\text{n}}}$ (2)

with S being the standard deviation and n the number of experiments (in this work it is 3).

**Confirmation of hydrogen peroxide generation**

Hydrogen peroxide generation was confirmed via two complementary methods. First, a colorimetric titration assay of the post-electrochemical oxygen reduction reaction (ORR) electrolyte was performed, with o-tolidine as the redox indicator. Chronoamperometry at -0.3 V vs Ag/AgCl for 30 minutes in O_2_-saturated 0.1 M KOH was employed to compare the peroxide production for each imprinted electrode. Following chronoamperometry, 1 mL of 1 M HCl was added to 2 mL of the KOH electrolyte to create an acidic environment for the o-tolidine reaction. Subsequently, 0.2 mL of 0.94 mM o-tolidine solution was added to 1 mL of the acidified electrolyte and allowed to react for 30 minutes. The absorbance of the resulting yellow solution was measured using a UV/Visible spectrophotometer, with the peak at ~436 nm indicating H_2_O_2_ formation. In a second approach, adapting the procedure of Fuku et al., ^1^ 500 μL of each electrolyte was mixed with 450 μL of 1 M HCl and 50 μL of 0.1 M FeCl_2_ in 1 M HCl. The absorbance of the resulting solution was measured using a UV/Visible spectrophotometer. The appearance of a peak around 335 nm, attributed to the oxidation of Fe^2+^ to Fe^3+^ by H_2_O_2_, further confirmed the presence of peroxide.

**Synthesis of chiral-encoded mesoporous Pt-Ir electrodes**

For the synthesis of the chiral-encoded mesoporous Pt-Ir electrodes (Fig. S16), gold-coated glass slides are first pre-treated by sonication in isopropanol and Milli-Q water for 30 minutes each, to ensure a clean surface for subsequent deposition. Then, a plating gel containing 29 wt% metal precursors (a mixture of H_2_PtCl_6_·6H_2_O and H_2_IrCl_6_·6H_2_O), 29 wt% Milli-Q water, 42 wt% Brij® C10 and a tailored amount of chiral template molecules is prepared. The chiral-encoded Pt-Ir is subsequently generated by electrodeposition on the pre-treated gold electrode from this gel at 40 °C at a potential of -0.05 V vs Ag/AgCl with the desired injected charge density. Finally, the prepared electrodes are continuously rinsed with Milli-Q water to remove any residual surfactant and chiral template molecules trapped within the mesoporous structures and chiral cavities.

**Supplementary Figures**


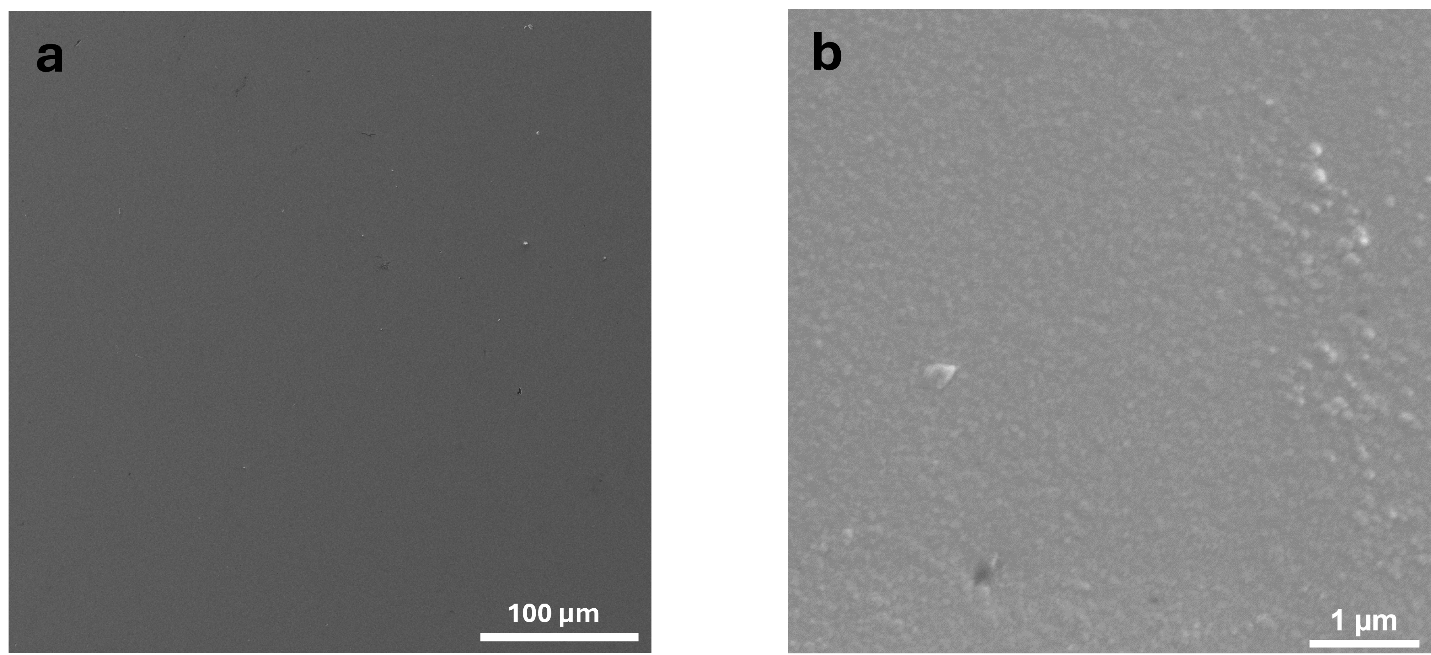


**Figure S1.** Top-view SEM images of a mesoporous Pt-Ir electrode at different magnifications a) scale bar 100 µm and b) scale bar 1 µm. These SEM images reveal a smooth surface morphology, indicating a successful and homogenous electrodeposition of a mesoporous Pt-Ir layer.^2^


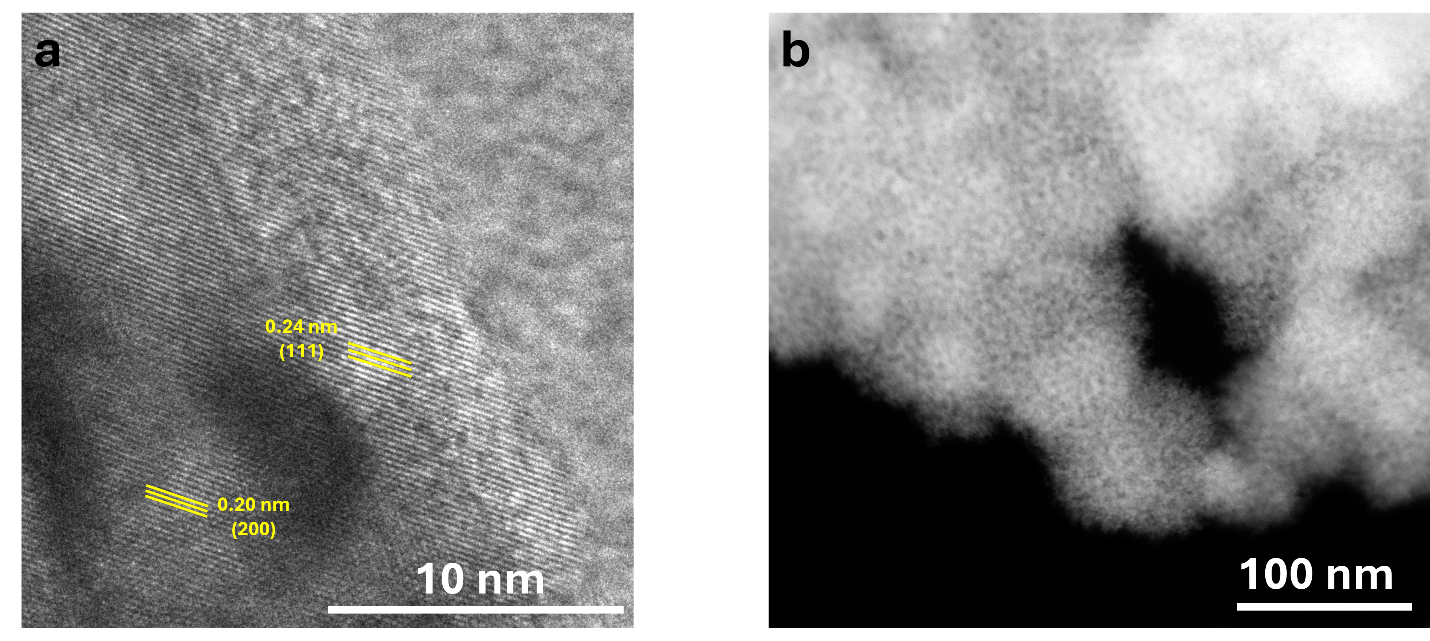


**Figure S2.** a) HR-TEM image of a Pt-Ir film (scale bar 10 nm) and b) TEM image of a mesoporous Pt-Ir electrode. The Pt-Ir film contains lattice fringes, with d-spacing values of 0.24 nm and 0.20 nm, matching with the (111) and (200) planes, respectively, being intermediate between the characteristic plane spacing of pure platinum and iridium, confirming the presence of the two metals in the mesoporous structure^3^. The TEM image reveals a mesoporous structure, analog to what has already been observed in previous work. ^4-9^


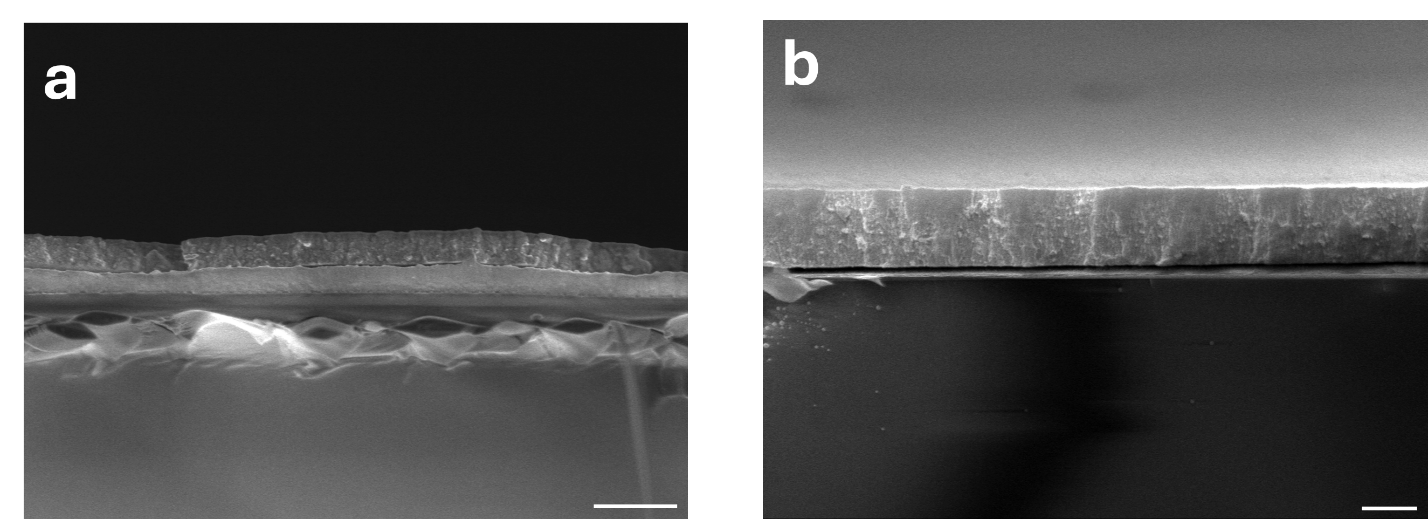
**Figure S3.** SEM images of the cross-section of electrodes with different thickness, generated by an injected charge density of (a) 4 C/cm^2^ and (b) 10 C/cm^2^ during metal deposition (scale bar 1 µm).


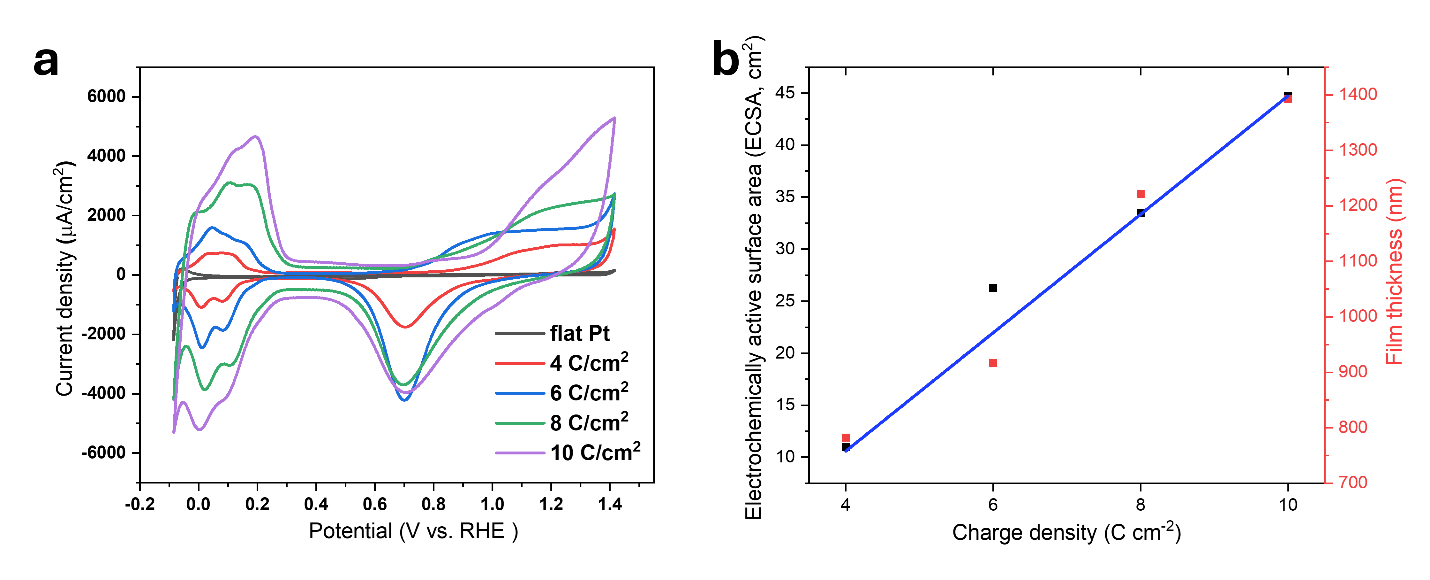
**Figure S4.** a) Cyclic voltammetry (CV) curves of electrodes with varying thickness in 0.5M H_2_SO_4_ solutions and b) plot of a comparison between the layer thickness, the electrochemically active surface area (ECSA) and the injected charge density during electrodeposition.

To assess the electrochemical features of these surfaces, we employed Cyclic Voltammetry (CV) for different non-imprinted mesoporous Pt-Ir electrodes prepared with varying injected charge densities, as depicted in Supplementary Figure 4a. The CV curves of the mesoporous Pt-Ir electrodes exhibit well-defined characteristic redox signals of Pt, especially the classic hydrogen adsorption and desorption features. Notably, the significantly higher area under the CV curve, compared to a commercial flat Pt electrode, indicates a substantially larger electrochemical surface area (ECSA) with an apparent roughness factor of approximately 210 ^10, 11^ at 8 C/cm^2^. This enhanced surface area, attributed to the presence of mesopores, is a highly desirable feature for efficient catalysis^12, 13^. These curves provide valuable insight into the electrochemical behavior and surface characteristics of the electrodes. When the charge density used in the electrode preparation increases (from 4 C/cm² to 10 C/cm²), the area under the CV curve also increases significantly. This observation directly correlates the higher deposition charge density with a larger ECSA as summarized in Supplementary Figure 4b. The ECSA of each electrode was determined via an analysis of the hydrogen adsorption peak, and plotted as a function of both, charge density and film thickness (calculated from SEM images in Supplementary Figure 3). As shown in Supplementary Figure 4b, both the active surface area and the film thickness increase linearly with the charge density injected during the electrodeposition step. These results illustrate the successful electrodeposition and indicate a mesoporous structure of the electrode layer.

To assess and compare the catalytic performance of the different electrodes, Linear Sweep Voltammetry (LSV) has been performed, as illustrated in Figure 2b of the main manuscript. The performance increase was calculated by using

$$\text{ }\left[ \frac{\text{j}_{\text{D}}}{\text{j}_{\text{L}}} \right]\times100 \%$$

with j_D_ and j_L_ being the peak current intensities of D-imprinted and L-imprinted electrodes, respectively. This gives, when using the j_D_ and j_L_ values of Figure 2b, together with the corresponding error bars:

$\text{ }\left[ \frac{74.40\pm8.41}{30.69\pm4.8} \right]\times100 \%$ = 242.4% ± 44.8%

**
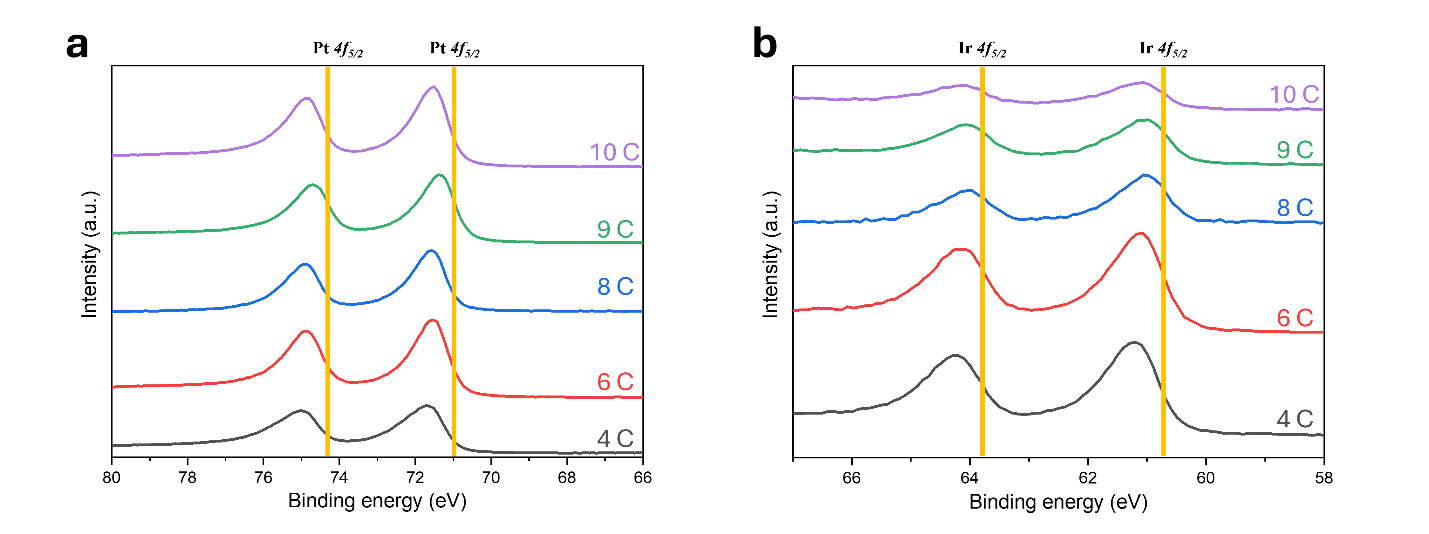
**

**Figure S5.** XPS signal for a) platinum and b) iridium, of alloy electrodes with varying thicknesses (4-10 C/cm^2^). XPS results show that standard monometallic Pt exhibits Pt 4*f*_7/2_ and Pt 4*f*_5/2_ peaks at 70.9 and 74.3 eV, respectively, while standard monometallic Ir shows Ir 4*f*_7/2_ and Ir 4*f*_5/2_ peaks at 60.8 and 63.7 eV (yellow vertical lines). Notably, the characteristic doublets of both Pt and Ir in the Pt-Ir films are shifted to higher binding energies. While the Pt peak shifts were consistent across all electrodes, the Ir peaks exhibited a more pronounced shift towards higher binding energies for lower deposition charge densities (higher relative Ir content).

**
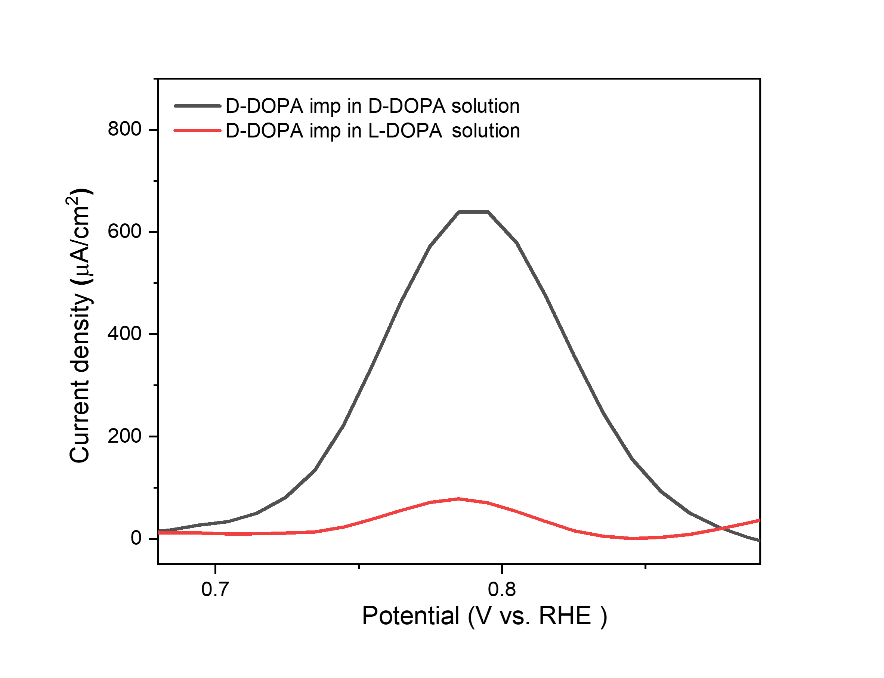
**

**Figure S6.** Differential pulse voltammograms (DPVs) of the electrooxidation of D-DOPA (black) and L-DOPA (red) recorded with D-DOPA imprinted Pt-Ir electrodes to verify the successful transfer of chirality to the Pt-Ir electrode. DPV is a sensitive electrochemical technique, well-suited for detecting subtle differences in the interaction between the electrode and chiral molecules.^5, 14^ The results indicate that the DPV signal is significantly higher when such an electrode is exposed to a D-DOPA solution than to a L-DOPA solution. This preferential response toward the imprinted enantiomer serves as solid evidence for the chiral character of the Pt-Ir structure.^5, 6, 15^


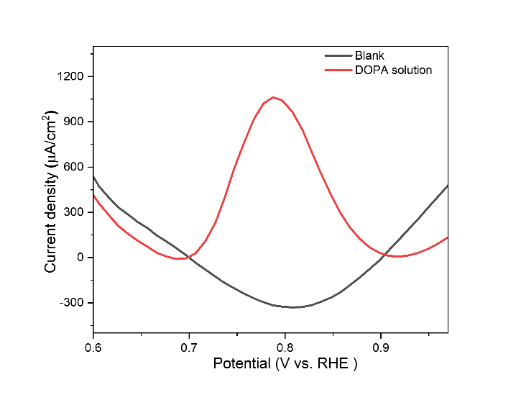


**Figure S7.** Differential Pulse Voltammograms (DPVs) of a fresh DOPA imprinted electrode, after overnight washing, in blank electrolyte of 50 mM HCl (black line) and in a 4 mM DOPA solution in 50 mM HCl (red line).


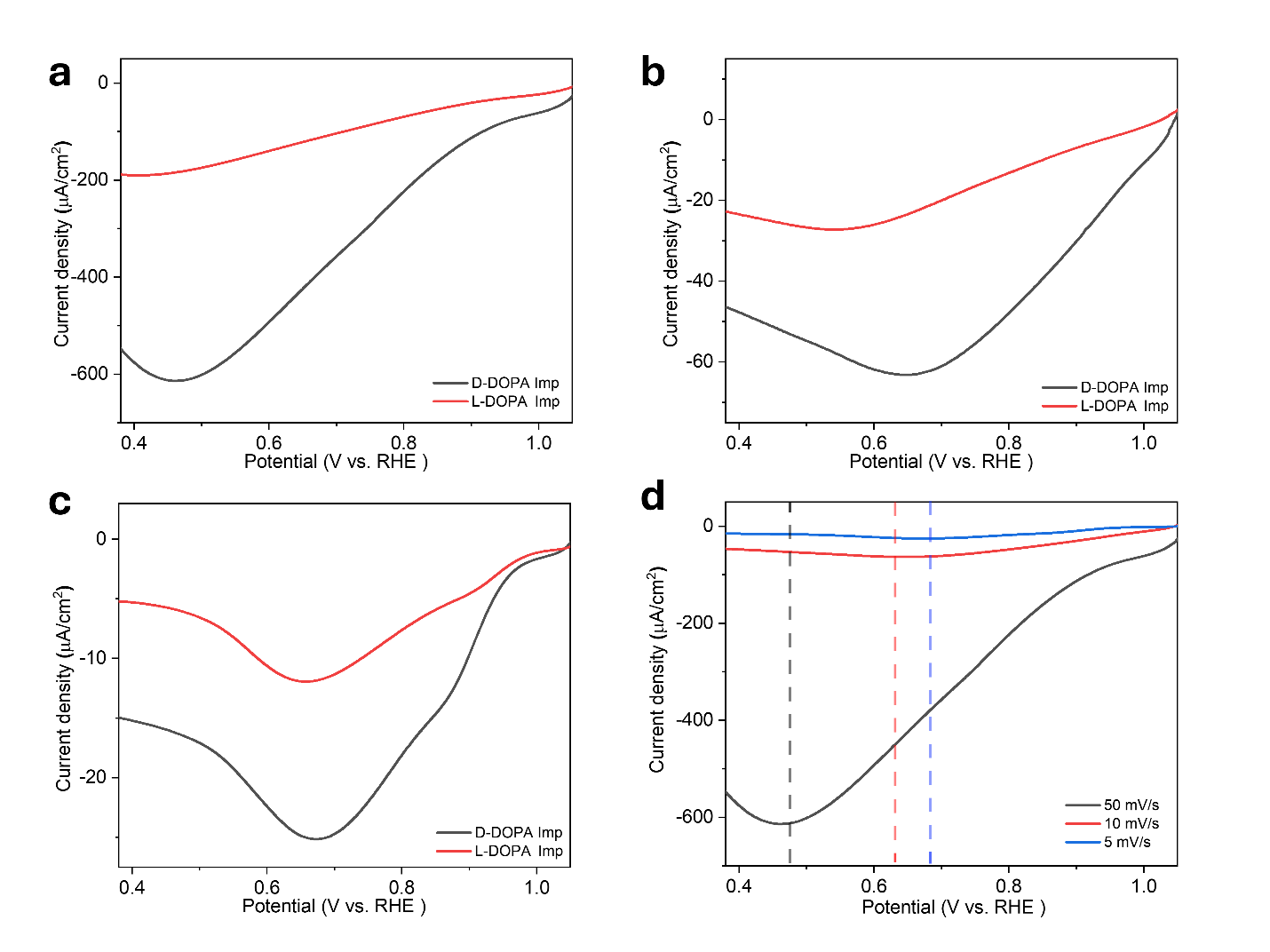
**Figure S8.** Linear sweep voltammograms (LSV) of D-DOPA and L-DOPA imprinted electrodes in oxygen-saturated solutions with varying scan rates a) 50mV/s, b) 10 mV/s, c) 5 mV/s and d) comparison of LSVs of D-DOPA imprinted electrodes at different scan rates.


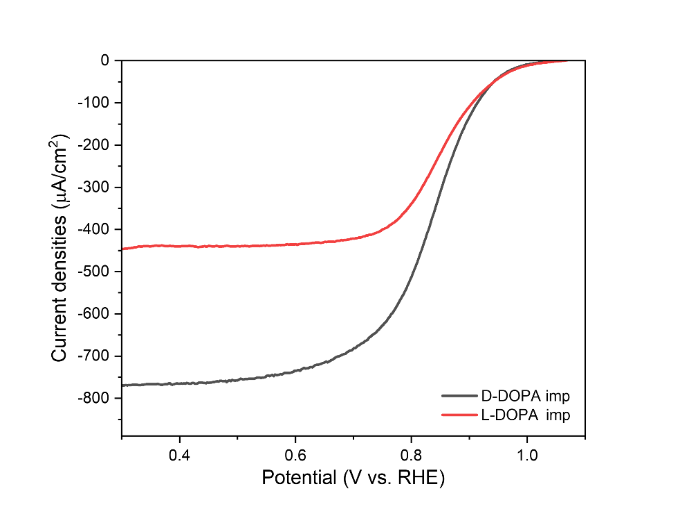


**Figure S9.** Linear sweep voltammograms (LSV) of D-DOPA and L-DOPA imprinted electrodes in oxygen-saturated solutions using a rotating disk electrode with rotating speed at 1600 rpm. The experiments are carried out under otherwise identical conditions as with the static electrodes.


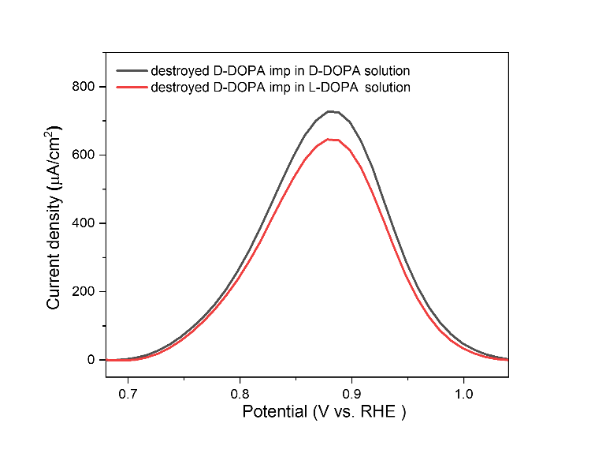


**Figure S10.** Differential pulse voltammograms (DPVs) of the electrooxidation of D-DOPA (black) and L-DOPA (red) recorded with an initially D-DOPA imprinted Pt-Ir electrode for which the chiral character has been destroyed by scanning the potential for 40 cycles from -0.1 V to +1.4 V vs RHE in sulfuric acid.


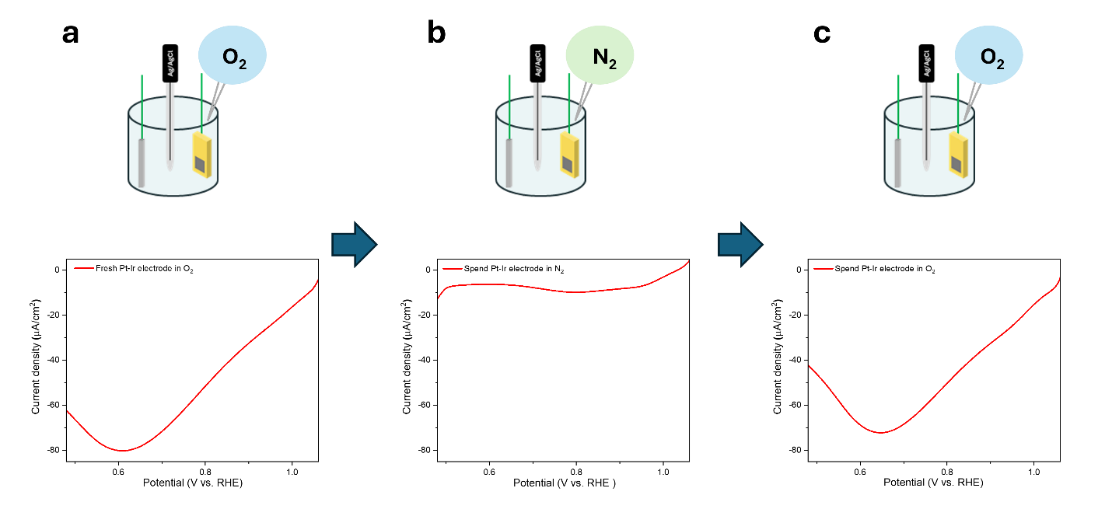


**Figure S11.** a) Electrochemical test of a D-DOPA imprinted mesoporous electrode for a) a fresh electrode in O_2_ saturated solution, b) an already used electrode in N_2_ saturated solution, and c) for a third use of the same electrode in O_2_ saturated solution.

**
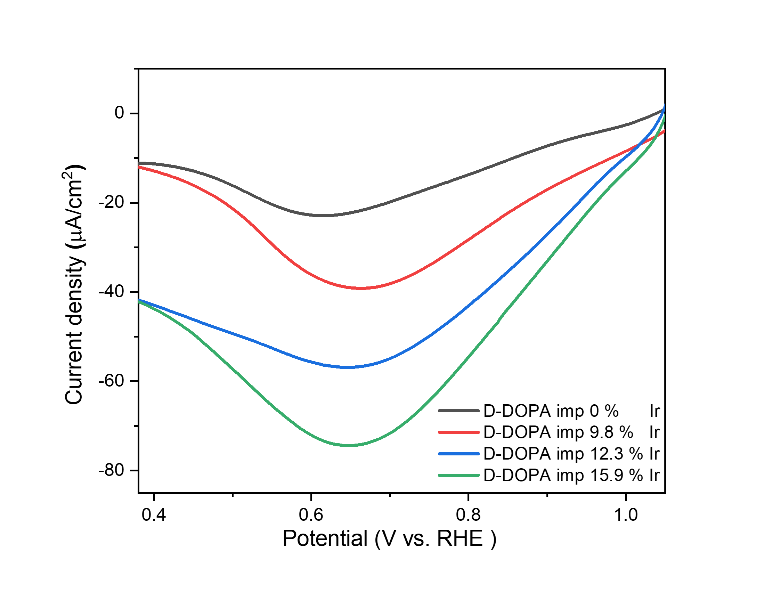
**

**Figure S12.** Linear sweep voltammograms (LSV) of D-DOPA imprinted electrodes at different percentage of Ir doping.

**
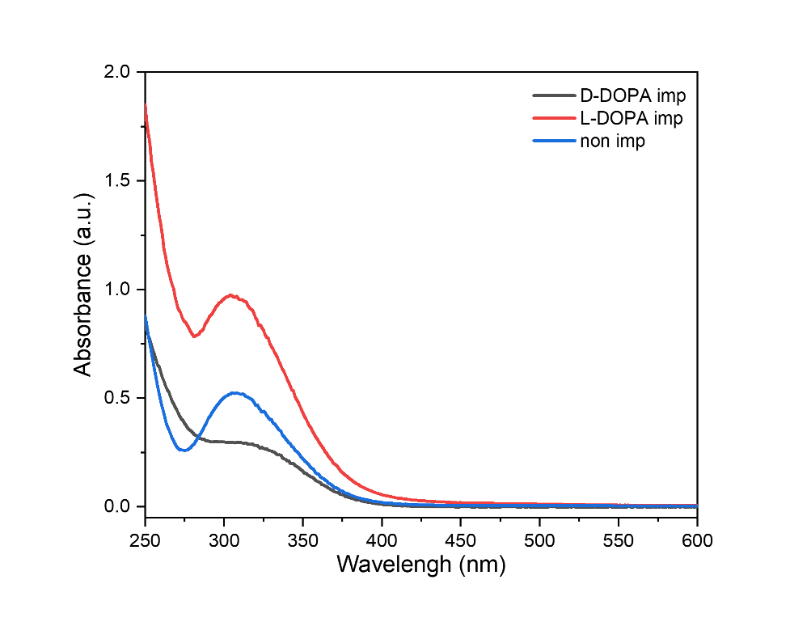
**

**Figure S13.** UV-Vis absorption spectra of a Fe^3+^ containing solution extracted from the electrochemical cell after chronoamperometric reduction of an oxygen-saturated solution at a potential of +0.66 V vs RHE, revealing a significantly lower concentration of Fe³⁺ when a D-DOPA imprinted electrode is used as a working electrode compared to L-DOPA imprinted or non-imprinted ones.


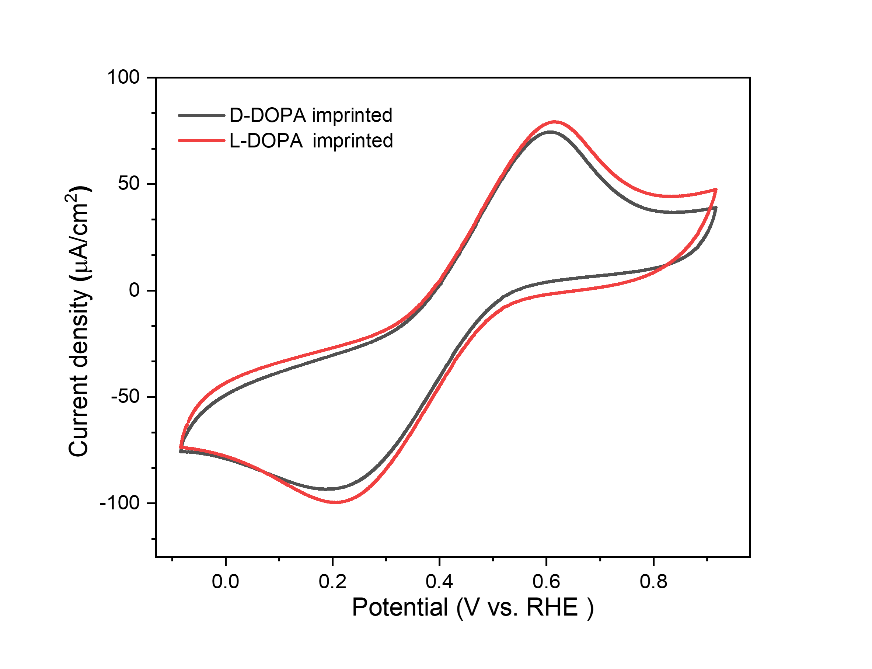


**Figure S14.** Cyclic voltammetry of D-DOPA and L-DOPA-imprinted electrodes in 50 mM K_3_[Fe(CN)_6_] and 0.5 M H_2_SO_4_.

***
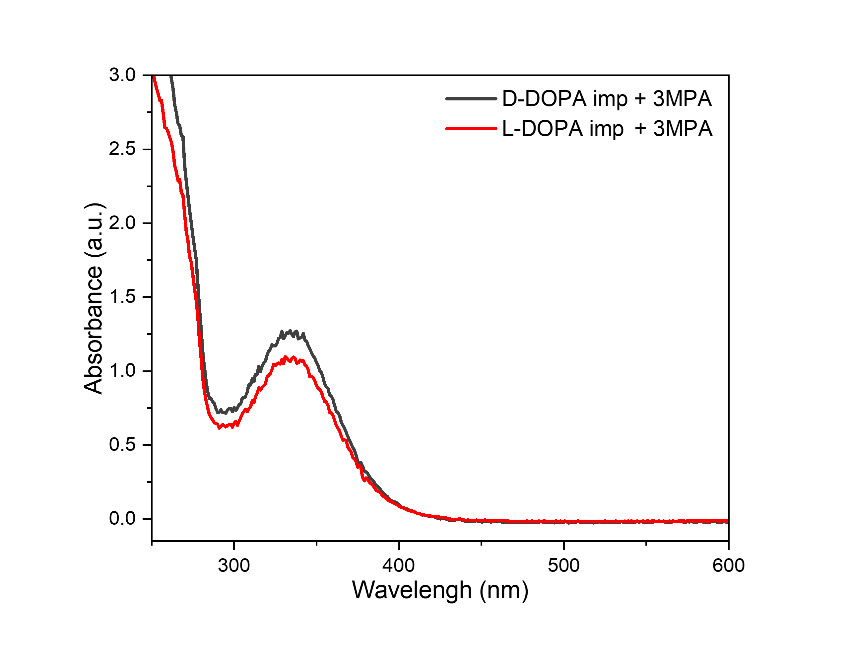
***

**Figure S15.** UV-Vis absorption spectra of a Fe^3+^ containing solution extracted from the electrochemical cell after chronoamperometric reduction of an oxygen-saturated solution at a potential of +0.66 V vs RHE, with a D-DOPA and a L-DOPA imprinted electrode, but both have been modified with a self-assembled monolayer of cysteine. This prevents the adsorption of oxygen and therefore the type of spin orientation transmitted through the chiral matrix has no longer an impact on the reaction pathway (2e versus 4e oxygen reduction). Consequently the same amount of hydrogen peroxide is produced with both electrodes.


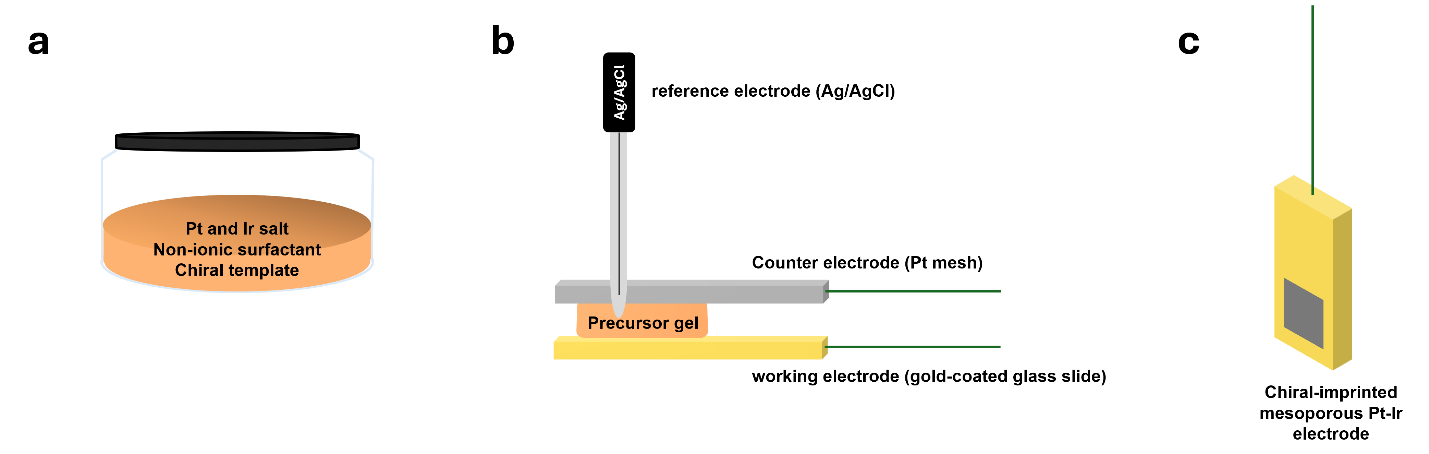


**Figure S16.** Schematic representation of the steps for the synthesis of a chiral imprinted mesoporous Pt-Ir electrode. a) Mixing of the precursors. b) Electrodeposition process. c) Final electrode after washing out the molecular templates.

**Table S1.** Nominal platinum-iridium ratios based on precursor masses compared to actual ratios determined by XPS measurements Quantitative analysis revealed a decrease in iridium content with increasing charge density/film thickness, attributed to the limited Ir precursor concentration in the plating gel.^6^

| **Based on the measured masses of the platinum and iridium precursors (at%)** | **Derived from the XPS measurements (at%)** |
| --- | --- |
| 100:0 | 100:0 |
| 90:10 | 90.2:9.8 |
| 85:15 | 84.1:15.9 |
| 80:20 | 87.7:12.3 |
| 75:25 | 79.8:20.2 |

**References**

1. K. Fuku*, et al.*, *ChemistrySelect* **2016**, *1*, 5721-5726.https://doi.org/10.1002/slct.201601469

2. D. Stoychev*, et al.*, *Mater. Chem. Phys.* **2001**, *72*, 360-365.https://doi.org/10.1016/S0254-0584(01)00337-6

3. F. Wu*, et al.*, *Surf. Coat. Technol.* **2004**, *184*, 24-30.https://doi.org/10.1016/j.surfcoat.2003.10.051

4. S. Assavapanumat*, et al.*, *Angewandte Chemie International Edition* **2019**, *58*, 3471-3475.https://doi.org/10.1002/anie.201812057

5. C. Wattanakit*, et al.*, *Nature Communications* **2014**, *5*, 3325.10.1038/ncomms4325

6. S. Butcha*, et al.*, *Nature Communications* **2021**, *12*, 1314.10.1038/s41467-021-21603-8

7. S. Assavapanumat*, et al.*, *Chemical Communications* **2019**, *55*, 10956-10959.10.1039/c9cc05854k

8. S. Butcha*, et al.*, *Chem. Commun.* **2022**, *58*, 10707-10710.10.1039/D2CC02562K

9. S. Assavapanumat*, et al.*, *Chemistry – An Asian Journal* **2021**, *16*, 3345-3353.https://doi.org/10.1002/asia.202100966

10. A. Kucernak*, et al.*, *Chem. Eng. J.* **2003**, *93*, 81-90.https://doi.org/10.1016/S1385-8947(02)00111-0

11. S. A. G. Evans*, et al.*, *Anal. Chem.* **2002**, *74*, 1322-1326.10.1021/ac011052p

12. Y. Ding*, et al.*, *J. Am. Chem. Soc.* **2004**, *126*, 6876-6877.10.1021/ja0320119

13. H.-W. Liang*, et al.*, *J. Am. Chem. Soc.* **2013**, *135*, 16002-16005.10.1021/ja407552k

14. S. Arnaboldi*, et al.*, *Chem. Sci.* **2015**, *6*, 1706-1711.10.1039/C4SC03713H

15. S. Assavapanumat*, et al.*, *Journal of the American Chemical Society* **2019**, *141*, 18870-18876.10.1021/jacs.9b10507
