# Supplementary material for: Chiral‐Encoded Pt‐Ir Surfaces as Apparent Spin Filter for Enhanced Oxygen Reduction
Source: Adv Sci (Weinh). 2026 Apr 10;13(36):e75175. doi: 10.1002/advs.75175 (PMC13317554; doi:10.1002/advs.75175)
Supplement: Supplementary file 2 — Supporting File 2: advs75175‐sup‐0002‐SuppMat.docx. [file ADVS-13-e75175-s001.docx]

**Reply to the referee’s comments concerning the manuscript “Chiral-Encoded Pt-Ir Surfaces as Apparent Spin Filter for Enhanced Oxygen Reduction” by Pasom et al (Ref. 8591018)**

***We would like to thank both referees for going again carefully through our manuscript and for the helpful additional comments and suggestions. We have taken them into account to prepare the second revised version and hope that this will allow the acceptance of our contribution for publication. We address below more specifically the individual items.***

**Reviewer 1 Comments to the Author**

The authors have addressed all my comments, and the revised manuscript is ready for publication in the journal.

***We are very happy to read that this referee is satisfied with the extensive revisions that we performed in the first round and that he/she now recommends publication without further changes.***

**Reviewer 2 Comments to the Author**

This work has been significantly improved after the authors carefully addressed the reviewers’ questions and concerns. However, a couple of questions still need to be addressed before publication.

***We sincerely appreciate that the referee recognizes the important efforts that we made in the first round of revisions to improve the manuscript. We address the remaining minor issues in the following.***

1. Determination of spin polarization using mc-AFM measurements or other instrumentation is still recommended even though the authors have decided not to measure it at this point, but it will be good evidence for future work if it is indeed not available to the authors now.

***We thank the referee for not insisting on these rather complex measurements, and we completely agree that mc-AFM experiments should allow providing further evidence for the CISS effect with these metal layers. We will actively work in this direction in the frame of future follow-up experiments.***

2. Figure 2b should include cyclic voltammetry (CV) results under oxygen-free conditions for the different chiral electrodes to avoid discrepancies in catalytic performance caused by variations in platinum loading. It is recommended that CV testing be conducted simultaneously with LSV testing for each electrode to eliminate interference from platinum content.

***We perfectly agree with the referee’s comment that one has to be very careful that the potentially slightly different platinum loadings on the individual electrodes do not lead to a wrong interpretation of the LSV results. This is exactly the reason why we systematically perform with ALL electrodes after the LSV experiments a cyclic voltammogram in sulphuric acid to measure their active surface area (and therefore the platinum content). This allows us to renormalize all the currents measured with LSV by the active surface area and present our data in the form of current densities, so that fluctuations of Pt loading are no longer influencing the presented results.***

3. What is the advantage of the proposed chiral catalyst in comparison to the previous chiral organic layer? For instance, stability and recyclability. It is recommended to provide additional experimental data to demonstrate the stability of the chiral electrodes.

***We thank the referee for raising this important point. Such studies have already been carried out by us in a very detailed way in a previous publication (***[***Nat Commun 12, 1314 (2021)***](https://www.nature.com/articles/s41467-021-21603-8) ***) and allowed to conclude that the chiral imprinted Pt-Ir electrodes are extremely stable, even under very harsh oxidizing conditions. They do not degrade even when cycling several times the potential to quite positive potential values. Their stability is considerable higher than the one observed for pure platinum, and this is exactly the reason why we have chosen these alloy electrodes for the present study. The stability is also again illustrated in the present contribution e.g. via the control experiments of Figure S11. The same electrode has been used several times under different conditions (with and without oxygen), showing only a minor loss of catalytic activity. This demonstrates, as requested by the referee, the stability and recyclability of these electrodes, which is certainly one of the potential advantages compared to previously studied systems based on chiral organic layers that are more fragile due to gradual desorption. We insist on this aspect now more clearly in the revised version of the manuscript. An additional and certainly most important advantage of the mesoporous metal electrodes lies in the fact that only when oxygen can directly interact with the metal surface it is possible to break the symmetry of the system, as demonstrated with the control experiments of Figure 4. The presence of an organic layer prevents such an adsorption, and consequently the original dual enhancement/inhibition feature completely disappears (Figure 4c).***

4. The manuscript should include LSV curves of chiral L/D/Rac DOPA electrodes from ORR tests at various Pt/Ir ratios in Figure 3a.

***We perfectly understand the referee’s request and have added a subset of the LSV curves corresponding to Figure 3a as a new figure in the supporting information (new Figure S12).***

5. Minor issue: many figures are difficult to read, for example, Figure 3. The quality of figures needs to be improved.

***We apologize that Figure 3 was difficult to read and we suppose that this is due to the conversion from Word to PDF format. We nevertheless increased now the overall size of Figure 3 and also of Figure 2 which was equally difficult to read.***
